# Robustness and disturbances in public transport

**DOI:** 10.1007/s12469-022-00301-8

**Published:** 2022-06-04

**Authors:** Liping Ge, Stefan Voß, Lin Xie

**Affiliations:** 1 Institute of Information Systems, Leuphana University Lüneburg, Universitätsallee 1, 21335 Lüneburg, Germany; 2 Institute of Information Systems, University of Hamburg, Von-Melle-Park 5, 20146 Hamburg, Germany

**Keywords:** Public transport, Resilience, Disturbances, Robustness, Delay management, Digital transformation, Bus bridging

## Abstract

Network-based systems are at the core of our everyday life. Whether it is electronic networking, electricity grids or transportation, users expect the networks to function properly and provide a feeling of safety and security. However, there may be disturbances. In this paper, we consider disturbances in the context of public transportation. The focus in this respect is on public transport planning and operations. To classify and cope with disturbances, one can find many ideas, including robustness, resilience, vulnerability, disruption mitigation or delay management. We survey related streams of literature and put them into perspective. As a major insight we show that different strands of literature exist that may benefit from becoming better connected and intertwined. Together with recent advances in information technology and solution methods, more integrated problem settings incorporating robustness and disturbances can play a major role in future planning and operations.

## Introduction

Transportation is at the core of enabling people’s everyday life. Usually, the first distinction in transportation refers to whether we move freight or people. In *public transport* (also known as public transit or mass transit) we are dealing with systems intended towards moving people. Nowadays, most of these systems, opposite to motorized individual transport or private transport, are operating passengers (single or groups) from the general public. Transport is from some origin to some destination, in most cases on a scheduled basis, with given routes to be adhered to. Deviations from these ideas and other types of concepts are possible to transport specific groups of people (e.g. *demand-responsive transit* especially for elderly or handicapped people etc.) or to enhance most notably mass transit systems with individualized solutions. We also envisage *Mobility-as-a-Service* (MaaS) describing an attempt towards mobility provided as a service rather than specifying the use of a specific mode of transportation upfront. This is enabled by combining all different types of transportation services under one umbrella (or, equivalently, account, access mode). In an era of autonomous vehicles becoming more and more available, this will be advanced over time, too.

Like many other systems, also transportation systems are prone to *error* or *disturbances* (that is, something is happening beyond the usual way of operation). In public transport one may react once a disturbance happens, and one may also take some measures upfront; this may be *reactive* or *proactive*. One may investigate, e.g., whether a public transport system is *vulnerable* or *robust* towards disturbances, i.e., whether it can be strongly affected or whether it can compensate them to some extent. That is, *robustness of a system* is the ability to keep up its functionality under conditions that deviate from their normal state (see, e.g., IEEE 1990; Cats 2016). Moreover, *recovery* of a system is the process of its salvage if it was disrupted or simply the process of bringing the system back to its original status after a disturbance or disruption. Often *resilience* is used to describe the ability of a system to withstand changes (see, e.g., Hosseini et al. 2016; Mudigonda et al. 2019; Wan et al. 2018), which is coping with the above-mentioned *vulnerability* referring to as the question to which extent especially extreme events can disrupt transport systems. That is, different concepts (and even words) are used when dealing with disturbances.

In this paper, we are mostly interested in disturbances in public transport (planning and operations) as well as related issues of robustness. Suitable means to cope with disturbances include robustness (as well as related concepts and methods). In literature, as indicated below, one can find many additional descriptions, phrases, or words, sometimes for the same things, including, but not restricted to, delay management, recovery actions, vulnerability, mitigation strategies, etc. Some of these concepts are used to analyze situations (e.g. bus bunching, where we observe two or more buses of the same line following close to each other without that being planned deliberately) while others are used to describe frameworks for solving problems (e.g. bus bridging, where buses are used to replace broken connections in other systems like metro or (light) rail). This needs differentiation. That is, we aim to discuss these different settings of disturbance, robustness etc. as well as their consequences and provide a survey of respective references. Moreover, we review some modeling/solution attempts from literature to explain possible consideration of integrated problem settings as well as an enhancement by means of incorporating robustness. Our choice of problem settings and references aims to shed some light on important issues to be explored further in future research. As a result, researchers as well as practitioners may benefit from a survey on these topics as well as a clarification of how the same or similar concepts are found under different names, allowing them to enhance the base of the literature to be considered. Moreover, advances in modern information and communication technology as well as related solution methodology and solvers, especially in the last ten years, allow to consider richer problem settings than what was possible in previous decades. This allows to consider disturbances and robustness at a level that has not been achieved before so that a survey in this respect seems beneficial.

The remainder of this paper is structured as follows. In the next section we sketch a few basic concepts focusing on public transport planning and operations, disturbances and robustness, worth being explored to set the pace. Section 3 clarifies differences regarding the timeline, i.e., distinguishing issues happening before and after a disturbance. Section 4 sketches solution approaches in generic terms. Section 5 gives a problem-oriented survey on different concepts to cope with disturbances, again focusing on public transport planning and operations. This includes the discussion of various problems as well as examples on how they are viewed in the light of disturbances. A summary of some case studies from literature is provided in Section 6.^1^ We conclude and provide some ideas for future research.

## Basic concepts

Before considering disturbances and a simple way to classify them, we provide some background about public transport. We also discuss some concepts like robustness in more detail. Finally, we resort to information management issues and a few measures of network connectivity as they are very often adhered to when dealing with robustness in public transport.

### Public transport

**Fig. 1:**
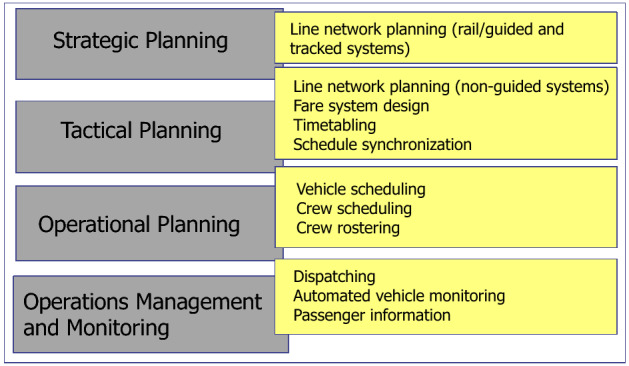
Planning stages in public transport

Classical planning problems in public transport may be structured along the timeline, i.e., strategically, tactically and operationally. Some selected basic understanding of public transport issues, especially regarding planning and operations, is summarized below using (Daduna and Voß 2000; Desaulniers and Hickman 2007; Ceder 2015; Vuchic 2005). Figure 1 describes the different planning stages in public transport following Daduna and Voß (2000, p. 8), Daduna and Voß (1996).

We assume that a *line* describes a consecutive number of stations that are served by means of a public transport vehicle (bus, train, etc.) running between these stations. The first and the last station are called, e.g., endpoint or terminus. *Headway* (or *frequency*) is used to express the distance between vehicles in a transit system measured in time or space (sometimes also called *cycle time*). The *dwell time* of a vehicle at a station is the time it spends without moving.

On a strategic level, decisions have to be made regarding lines to be offered. This is most demanding, if expensive infrastructure needs to be installed like for guided and tracked systems (e.g. railtracks and train stations). This is easier if a bus line is set up and it “just” needs some sign posts or some bus stations (of course it also needs investments in buses, drivers need to be hired, etc.).

Several lines together build a *public transport network*. Within such a network, there can be different modes of transportation. Concerning the use of multiple modes like, e.g., rail, bus and ferry altogether in a public transport network there can be different levels or a multi-level network. That is, a *multi-modal network* is a network integrating multiple modes of transportation. Moreover, a level in a public transport network refers to a specific degree of detail of a service, i.e., (inter)regional level, metropolitan level and local (urban) level. The integration of several levels in a network is called *multi-level network*. Integrating several modes can be called multi-modal or vertical integration and the integration of several levels (within the same mode) can be called multi-level or horizontal integration. Deviating from this simplified definition could be necessary, if local areas with different structural designs are concerned (e.g. the use of large articulation buses on arterial streets versus small buses in narrow street districts). Note that these definitions are motivated by the work of Yap et al. (2018) as well as our work in maritime shipping (Shi and Voß 2007; see also Daduna 2020). For a survey on optimization problems in line (network) planning see, e.g., Schöbel (2012).

Once a line network is set up, one has to think about pricing (like fare zone design) and alike.^2^ On the tactical level one also refers to setting up a timetable with all its specific considerations, like, e.g., the coordination of different lines so that schedules are synchronized to allow for possible transfers of passengers. The timetable has to be fulfilled, so appropriate plans have to be made for transport vehicles (buses, trains), related personnel (e.g. crew, drivers, conductors). Making specific plans is usually called vehicle scheduling, crew scheduling, duty scheduling and duty rostering, where the following definitions are adhered to. A  *roster* or a *schedule* is a list of personnel and associated information, e.g. location, working times, responsibilities for a given time period like a week, a month or a holiday season. That is, a duty roster is a schedule which assigns tasks, shifts (e.g. the day shift or a night shift), and away missions to crew members. Being aware of the different planning stages, it is important to do things in a certain (not necessarily hierarchical) sequence. For instance, given a timetable, in vehicle scheduling vehicles are assigned to specific trips that need to be performed; the latter may be called *vehicle blocks*. Once a sequence of tasks in a vehicle block is defined, those tasks should be assigned to a duty for a certain period, like a morning shift or 5 h or a working day. These duties need to follow given regulations and policies like length of work without break etc. The process of defining duties is often called *crew scheduling*. Breaking it down to dispatching and operations management (see Fig. 1) also relates to specific final parts of the planning stages like giving a specific driver a specific schedule (described as a roster above) and then, within a schedule, putting the driver on a specific vehicle. A comprehensive survey of related optimization problems can be found, e.g., in Schöbel (2006). A literature review focusing on bus systems is Ibarra-Rojas et al. (2015).

Recent tendencies in the transition between tactical and operational planning include the joint planning of different aspects like the integrated vehicle and crew scheduling problem (see, e.g., Mesquita et al. 2009, 2013; Amberg et al. 2019; Lin et al. 2020). That is, problem settings become more and more rich,^3^ incorporating problem-specific aspects observed in practice. For instance, starting from the classical vehicle and crew scheduling problem, one may include additional legal constraints, company-based policies (like day-off patterns in Mesquita et al. (2013)) or even robustness (e.g. by means of fixed buffer times and/or delay propagation measures as in Amberg et al. (2019) and Ge et al. (2022)).

Once the named as well as related problems are solved (heuristically or exactly) and corresponding plans are available, they need to be put into action (or operation). This refers to running the system including dispatching, monitoring vehicles and alike. Moreover, related information management issues need to be considered like, e.g., providing passenger information.^4^

Finally, we note that research in public transport can vary along the timeline but also be related to the perspective and possible modeling approaches as well as the modeling scale. The latter especially relates to the level of detail. On a very detailed level we have *microscopic* models, on a broader scale we consider *macroscopic* models. As an example, in a macroscopic model we assume that we have a very abstract problem representation where, e.g., a station is just a node in a graph or network while a microscopic perspective considers the detailed tracks etc. within the station. In a *mesoscopic* model both concepts, microscopic and macroscopic, are combined in the sense that they include elements from both models, i.e., some parts are modeled with a lot of detail, while others are not (sometimes implemented as or called stub modules).

Beyond these views we may also consider a *microeconomic* view on public transport as is usually found in transport economics. This realm could focus on the involved stakeholders and their resources. These are, namely, operators with their means of transport plus the available infrastructure and passengers or users with their demand, time etc. Often we see a distinction regarding the latter considering access, egress, waiting and in-vehicle time. Transport economics then accounts for the (intended or assumed) demand and implies measures like the number of vehicles as well as incurred frequency and cycle times; see, e.g., Jara-Díaz and Gschwender (2003). Note that this discussion may be extended by policy-oriented means of assuring a minimum level of services of general interest and welfare provisions.

### Disturbances

While the planning stages described in the previous subsection have been put into practice or are put into practice, there certainly are or will be *disturbances*. As mentioned before, a disturbance is something happening beyond the usual way of operation. It can be any type of trouble, fault, disorder, disruption, impairment, interference, damage, harm, agitation, uneasiness, etc. related to public transport.

**Table 1:** Disturbances: classification dimensions and examples

Dimension	Specification	Examples
Planning	Planned	Maintenance, labor strike, expected (e.g. sports) event
	Unplanned	Traffic jam, abnormal events (severe weather-based accidents, terrorist attack), absence of drivers due to illness, crew shortage, rolling stock breakdown, crowding, unexpected event
Probability	High	Demand fluctuation
	(Very) low	Terrorist attack, pandemic with lockdown or curfew
Impact	(Very) low	Broken escalator
	(Very) high	Terrorist attack
Time: occurrence	Pre-trip	Forecasted storm
	En-route	Tree fallen over on tracks
	Time of the event	Peak, off-peak, day, night, etc.
Time: duration	Short	Fixed versus estimated versus unknown duration
	Long	Fixed versus estimated versus unknown duration
Type	Natural	Weather-based (e.g. closed street due to flooding)
	Man-made	Illegal parking with blocking of public transport vehicle
Scope	Local	Limited spatial impact
	Regional/global	Severe weather
Location	Inside/internal	Within the public transport system itself (e.g., a bus breaks down)
	Outside/external	An external influence (like a road closure)
Frequency	(Very) seldom/non-recurrent	Terrorist attack, pandemia
	Often/recurrent	(Delay due to) traffic jam
		Restricted availability of infrastructure
Miscellaneous		Psychophysiological (driver stress)
		Near accident, pronounced fatigue
		Correlated events (clear ice, accident)
		Complexity

For coping with disturbances, we start with a hands-on classification of disturbances in public transport. Obvious dimensions, as presented in Table 1 (which is based on a brainstorming effort of the authors; see also, e.g., Yap (2014) for a list of possible disturbances in public transport), are related to the distinction of planned disturbances (like in case of planned maintenance and repair) while others come more or less as a surprise (like unplanned accidents, congestion or just a delay); disturbances may be repetitive (viz. *recurrent*) or not (*non-recurrent*). Disturbances can be man-made or natural, they can be minor or severe, and much more. Disturbances are usually not known beforehand but occasionally they may be pre-planned (e.g. in case of a pre-announced strike (van Exel and Rietveld 2009) or in case of maintenance work; see, e.g., Sect. 5.8). Table 1 may be extended by additional topics (obvious as well as far-fetched ones). Let us take an example. When it comes to a disturbance of public transport that may be considered by means of a mathematical programming approach, mitigation may be considerably improved and people may be in favor towards or at least not be distracted from using public transport because of the disturbance. However, other types of disturbances may be beyond the impact of being treated by planning models (at least at first glance) like fare evasion (Barabino et al. 2020) or crime (Newton et al. 2004), but they may still have a major impact on the use of public transport such as the comprehension of fear or unfairness (like realizing that other people get the service for free without being discovered in case of fare evasion, or experiencing crime while using public transport).

Planned events like maintenance usually come with an advance notice while most disturbances are coming in an unplanned way. Exceptions are events that may to some extent be forecasted like, e.g., certain traffic jams during peak hours. When we speak about probability regarding an event to happen, we mostly refer to the likelihood of occurrence. Often this also goes in hand with characteristics influencing the possible impact of a disturbance, like whether it happens during peak or off-peak hours, whether it happens on a weekday or on a weekend, during school holidays, during day or during night, etc.

Disturbances cause *primary*/exogenous delays (also called source delays (Dollevoet et al. 2018)). Such delays usually cannot be prevented and are distinguished from *secondary* (or knock-on, propagated, reactionary) delays. Propagated delays may be caused by the delayed arrival of vehicles from previous duties or tasks, which use common resources. Propagated delays are those delays one can influence by making scheduling decisions, such as Amberg et al. (2019) and Ge et al. (2022) in public transit and Ionescu (2018) in the airline sector.

Based on different kinds of disturbances, one may distinguish implications based on the involved stakeholders, be it transit operators, passengers or even policy makers. In literature, mostly the first two are focused. For instance, taking a passenger-oriented view, one may define various measures characterizing a disturbance and its influence (sometimes called *robustness indicators*; see, e.g., Friedrich et al. 2017; Cats et al. 2017). Possible indicators could measure the disturbance-based delay of the passenger at the final destination. Exemplification includes the number of delayed passengers (eventually arriving with a delay larger than a given threshold at their intended destination), the total delay time (summing up all delay times of late arriving passengers at their destinations), the average delay time per affected passenger or the proportion of passengers who need to change the initially intended route to reach the final destination (within a certain time limit).

**Fig. 2:**
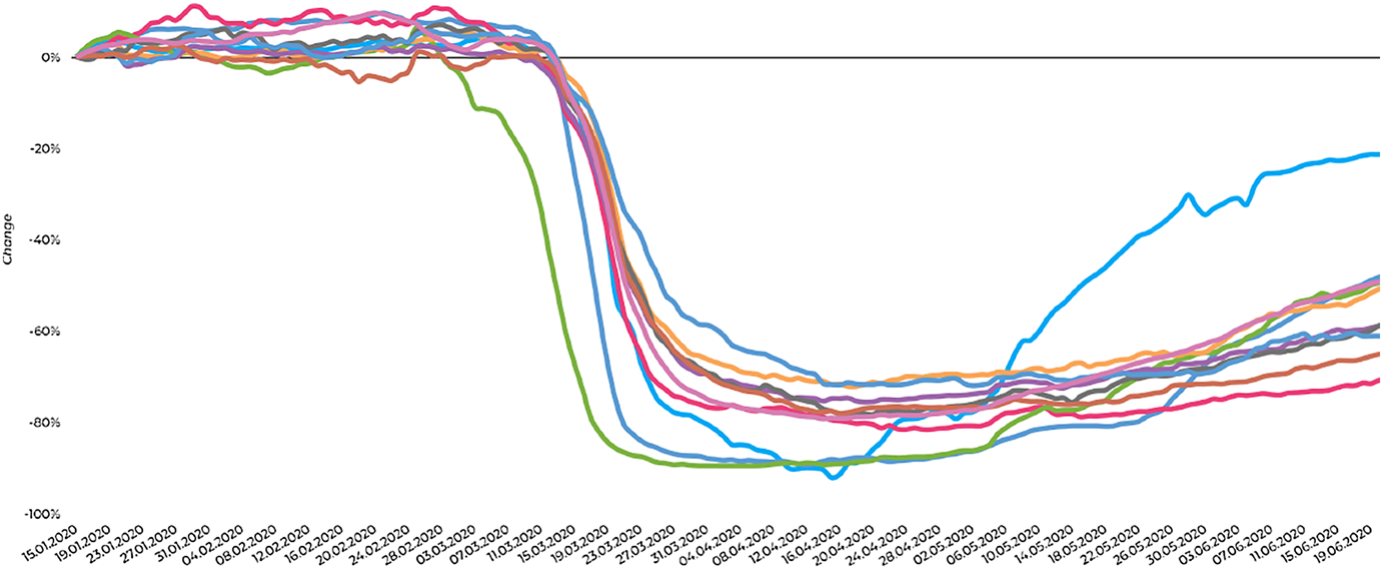
Decline of bus patronage worldwide; https://moovitapp.com/insights/en/Moovit_Insights_Public_Transit_Index-countries, last access June 19, 2020

The recent Covid-19 pandemic is a very-high-impact-very-low-probability disturbance. In times of lockdowns the operations of public transport (including bus, rail, ferry and taxi) have been suspended or at least been reduced considerably in many places; cf. Fig. 2.^5^ In many cases stations were temporarily closed or not served. For instance, in Wuhan, China, the municipal government expropriated, among others, bus stations to build shelter hospitals rather than using them for public transport purposes (Yu and Li 2020). Literally, the Covid-19 pandemic might deserve a comprehensive paper on its own; for some references see, e.g., Hirschhorn (2021), Marsden and Docherty (2021) and Mützel and Scheiner (2021).

**Fig. 3:**
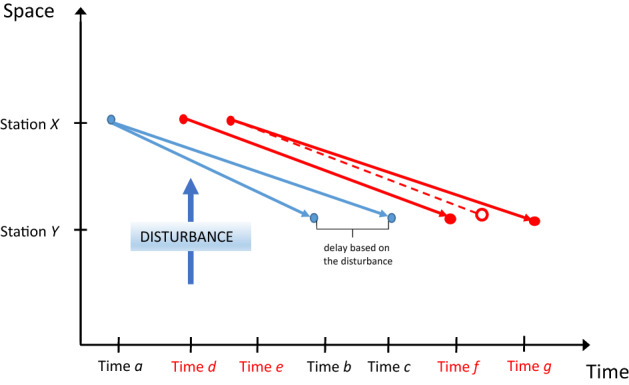
Time space diagram in case of load dependency (own elaboration)

Disturbances are not only unforseen events, they can also be expected events, eventually with an uncertain time of occurrence. As examples, consider weather-based disturbances with an uncertain but expected time of occurrence or the lifetime of a battery in battery-driven buses or vehicles that will eventually come to an end at some point in time (see Sect. 5.3 for more detail). Note that based on demand fluctuation as well as other factors, even a traffic jam could become an expected event. As an example, consider Fig. 3. Red arcs describe the situation where the trip from Station *X* to Station *Y* needs more time if it is starting at time *e* rather than time *d*. This could be related to different traffic conditions or speed limits at different times and in that sense be a foreseeable event, while traffic jams, in general, may not be always foreseen.

Some of the possible disturbances listed in Table 1 may be open for debate, especially when focusing on planning and operations, as they might arise only in the eyes of the beholder. Examples include psychophysiological issues like driver stress, near accidents or crowding. If reasonably designed, questionnaire-based studies in this area can be an important methodology as well as an issue requiring consideration (especially when measuring soft factors such as, e.g., happiness). As an example, consider crowding which usually is assumed to be an issue with difficulties in specification (Haywood et al. 2017). That is, if capacity is not counted by the number of seats, physical closeness between travelers per se is often not taken as a hard constraint (although this might be necessary). However, closeness may impact on customer satisfaction on a subjective level, e.g., considering social distancing or happiness in using public transport (see, e.g., Duarte et al. 2010 for the latter).

Performing integrated planning changes the degree of freedom but also increases the interlinking of different problems. This also means that disturbances in one dimension may more strongly influence those in others. Additionally, the integration may also concern the joint consideration of multi-level public transport networks. Then, the *exposure* or the impact of a disturbance may be defined (Yap et al. 2017). Taking earlier classification criteria, the exposure of a disturbance can be defined as the product of the frequency of the disturbance and the duration of the disturbance. Both values can be seen as probabilistic values, possibly being independent from each other.

Complexity can be used to classify problems regarding their hardness and the question whether to use heuristics or exact approaches (see Sect. 4). However, complexity can also mean the difficulties arising from and with disturbances. Once a disturbance (or disruption) occurs, one may encounter knock-on effects causing additional (multiple) disturbances; information availability on the disturbance, involved stakeholders and available resources may not be available; stakeholders may not behave in an expected way. An important lesson regarding complexity learned from Dekker et al. (2021) is the issue of when to consider centralized and when to consider decentralized decision making. Managerial treatments of disruptions are investigated in Piner and Condry (2017). As a major (well-known) conclusion, the provision of accurate and consistent information is most important (emphasizing the need for efficient passenger information and information management).

Considering existing literature, the cause of disruptions is one of the most important issues when differentiating between various concepts like robustness and vulnerability. In that sense, robustness and vulnerability are often used as opposites (Knoop et al. 2012). Simply spoken, *robustness* describes the strength of a network and *vulnerability* describes the weakness of a network. The next two subsections are devoted to these concepts and deepen their definition and understanding.

### Robustness

*Robustness* is the ability of a system to resist against adverse actions or events (to some extent). In different words, a system is called robust if it may cope with changes without the need to adapt. Using the idea of robustness describing the strength of a (public transport) network, definitions of robustness, not only in the public transport literature, seem to occur as a dime a dozen.^6^ In general as well as in public transport networks the idea of a network being fault tolerant (a different word for robust) is used. However, fault tolerance can have different dimensions and different stakeholders that may be influenced by related measures. Many of these measures are devoted to network connectivity (see Sect. 2.6).

In this respect we also find *reliability*; see, e.g., Soza-Parra et al. (2019). The common comprehension is that a public transport system is reliable if it can be trusted to work well or to behave in the way it is supposed to work. *Flexibility* may also be used in this respect. Coming more from an engineering point of view, it relates to bending or pushing a system without breaking it. Other words or synonyms are adaptability, openness, versatility, and adjustability. *Stability* of a plan relates to the degree to which it remains feasible under variations of the environment without the need of major modifications. In a business-oriented context one might not only strive for feasibility but also for cost-efficiency. Often these words are used in public transport without thinking too much of their specific meaning; the same seems also true in other industries. We refer the interested reader to a few references mostly focusing on the airline industry: Dück et al. (2012), Ionescu and Kliewer (2011) and Ionescu (2018). In the spirit of the above definition of robustness “the degree of sensitivity of various rescheduling algorithms to variations in process times (running and dwell times)” can also be addressed (Larsen et al. 2014) and be seen as a robustness measure.

Regarding robustness and solution approaches we need a more detailed understanding of what the strength of a system (or being immune against interference) really means and we refer to robust optimization and light robustness in Sect. 4. Beyond this notion we also find the consideration of *recoverable robustness* and *quasi-robustness* (Veelenturf et al. 2016b).

### Resilience and vulnerability

*Resilience* is an indicator for the ability of systems to withstand disruptions within acceptable degradation parameters but also their recovery time. Taking the Latin origin resilire of the word, one may define resilience slightly differently in the sense of the ability to bounce back from a disturbance. After analyzing the related literature, many references are devoted to the capability of systems being able to absorb disruptive events and to adapt accordingly. Moreover, recovery is implied to be a critical issue in resilience. For some general exposition on resilience and relations into supply chain resilience see especially Bababeik et al. (2018), Hosseini et al. (2019) and Hosseini et al. (2016).

In a transportation research-oriented editorial, Caschili et al. (2015) specify that resilience refers to the inherent capability of networks to adapt and return to normal conditions after some disturbance like a critical event. In that sense, they emphasize recovery as a major driver of resilience. On the other hand, according to the same source, *vulnerability* relates to the risk and probability that extreme events can disrupt transport systems. Once network planning tasks are performed and related management is undertaken, objectives are usually devoted towards maximizing network resilience as well as minimizing vulnerability. Most important in this respect is the preservation of connectivity, though this has to be specified in detail as there are various measures describing connectivity. Again, we refer to Sect. 2.6, where, among others, different graph-theoretic concepts are explained to measure connectivity (see, e.g., Derrible and Kennedy 2009) possibly indicating which networks might be more resilient or vulnerable than others.

In a similar spirit, Cats and Jenelius (2018) focus on the relation between network performance and the degradation of line or link capacities. By establishing a vulnerability curve, impacts of capacity reductions in a public transport network are assessed using a dynamic non-equilibrium model. Literally, the interplay of resilience and vulnerability of complex networks against failure (as a whole or related to its parts) goes back to classical operations research. Which components of a network need more strength, possibly expressed by means of redundancy (like in extending capacity), and where is a network potentially the weakest? In that sense, the targeted destruction of a network is concerned with the same components or constituents or similarly handled like the potential increase of its strength. The notion of attack vulnerability of complex networks goes back to studying transportation as well as computer networks clarifying where to add or remove nodes and/or links to make a network more resilient or vulnerable. Examples include Berche et al. (2009) and Jin et al. (2014). Taking related preventive measures to avoid attacks or at least reduce their impact is an important research area; see, e.g., Bruyelle et al. (2014).

The discussion about resilience vs. vulnerability in connectivity network structures is also reflected in Reggiani (2013) and Reggiani et al. (2015) (even including some considerations of scale-free networks). Going back to resilience, we may also define as follows (Jin et al. 2014, p. 17): “Resilience of a system refers to the ability to withstand disruptions within [an] acceptable reduction in service performance.” Implications may call for extended efforts to increase protective measures rather than investing in post-disruption recovery methods (see the bus bridging concept as an example). In the same spirit, several authors propose different ways to quantify resilience. For instance, D’Lima and Medda (2015) propose a certain measure and they use stochastic models in which one parameter is interpreted as the resilience. Their ideas are exemplified by means of the London Underground.

General surveys on resilience in transportation systems can be found in Wan et al. (2018) and Beśinović (2020). Another review is Mattsson and Jenelius (2015). They discuss various concepts of transport system vulnerability and resilience and review related research. As a major conclusion they highlight possible benefits of cross-disciplinary studies focusing on topological and system-based vulnerability. A recent collection of surveys focusing on network resilience, service reliability and disturbances can be found in Yap and Cats (2021), van Oort (2021) and Shalaby et al. (2021). They are especially important as they support our view on public transport planning and operations as a major driver of research and applications. Moreover, an interesting research issue relates to whether resilience uses aggregate or worst-case measures with respect to different stakeholders, a question comprehensively considered in Vodopivec and Miller-Hooks (2019). Moreover, the authors of the latter source investigate whether a bad comprehension of a public transport system relates to the system itself or the possible resilience measures in case of disturbances. In Reggiani et al. (2015) vulnerability and resilience in transportation science is reviewed, too. The focus, though, is slightly different as they view it from a connectivity and accessibility angle and relate it to robustness, reliability and friability. Considering vulnerability regarding major discrete events mostly refers to large, non-recurrent events which affect infrastructure availability. Links where the product of exposure to disturbances and the impact of these disturbances is highest are identified as most vulnerable by Yap (2014) and Yap et al. (2018). Related to this context are also maintenance and repair as well as methods to test and quantify the resilience of infrastructure components in any dimensions. An exemplification regarding railway signalling characteristics can be found in Simons (2019).

A great citation analysis focusing on resilience, vulnerability and alike can be found in Sugishita and Asakura (2020) and Sugishita and Asakura (2021). Other works on resilience with a transportation and public transport focus include Ren et al. (2019) and Cox et al. (2011).

### Information management

Information may be viewed as purpose-oriented knowledge, demonstrating action-determined knowledge of various conditions and developments in reality. We define *information management* as purpose-oriented provision, processing, and distribution of the resource information for decision support as well as the provision of respective infrastructure (Voß and Gutenschwager 2001). That is, information management is understood, among others, to be an instrument for making information distribution operable for an enterprise. In that respect, it becomes an enabler for efficient innovation management including digital innovation. Thinking in terms of core innovations, information technology may be seen as a core innovation with an omnipresent penetration throughout all other areas or industries (see, e.g., Voß and Gutenschwager 2001). The adoption of information management in public transport is well described and supported in Daduna and Voß (2000) and ever since used in an increasing manner. Beyond being an enabler for innovation, it is seen as an enabler for efficient planning and operations.

**Table 2:** Support regarding internal and external information management

	External/static	Internal	External/dynamic
Information usage	Information chain-related demand, purpose-oriented knowledge	Planning (data, information)	Dynamic information at stops, in vehicles, on mobile phones
Information systems	Timetable booklet, web-based information systems	Automated vehicle monitoring system, planning systems	On-board information systems, monitor walls, social media
Infrastructure	Clients, network	Server, GPS (global positioning system) network	On-board computer, pillar, network, telecommunication devices

Information distribution and data management are closely related to *real-time control*. Control, which is conducted in real time, can be implemented in various dimensions (see Ibarra-Rojas et al. 2015) to guarantee an efficient service during system operation. Examples include station control which aims at vehicle holding to improve service regularity or to ensure passenger transfers as well as inter-station control including speed control and the application of traffic-signal priority. In different words, actions are taken in real time given a disturbance (e.g. deviation from schedule adherence). That is, disturbances need to be detected and related measures need to be taken. Based on comprehensive data availability and data-driven technology being readily available in many cases, we often see traffic control centers that take the business of observing and controlling the daily operation as well as to take measures in case something unusual or unexpected happens, e.g. a disturbance. Modern information and communication technology can help to perform the necessary control measures and they need to be collected, e.g., in such a control center and its information systems. For a survey of state-of-the-art technology for automatic train operation, especially in urban rail systems, we refer, e.g., to Yin et al. (2017). The more this technology is used, the more one also needs to account for the relationship between different levels of automation and the robustness, reliability etc. of public transport networks. In that sense, the relationship between primary and secondary causes for disturbance reaches new levels of complexity due to being massively intertwined.

One important use of real-time control is to improve service quality provision. Exemplification can be to treat schedule synchronization and use it to ensure planned transfer adherence. This issue (schedule synchronization in particular as well as service quality in general) becomes even more important if we are concerned with changing headways and last trains or buses (Voß 1992; Daduna and Voß 1995; Kang et al. 2015; Wu et al. 2015; Yin et al. 2019 and Nesheli and Ceder 2014, 2015; Nesheli et al. 2017, where the contributions of the latter ones are very similar). Especially under those circumstances, the impact of disturbances may become much more severe than, e.g., in peak hours. While this ties in with delay management (see Sect. 5.4), it also goes beyond. Moreover, considering multi-level networks makes the problem more complex, especially if mobility-restricted user groups are considered. We should note that research incorporating transfers often works without consideration of schedule synchronization as, e.g., prevalent in Dakic et al. (2021). Raising the issue of *substitutability* might state that just walking reduces the number of transfers. However, a comprehensive consideration of substitutability may lead to multi-objective optimization problems considering different objectives like comfort, price, and time and the issue of related trade-offs. This can be seen in regard to synchronization issues as well as passenger information; see, e.g., Daduna and Voß (1995, 1996). Substitution can also be seen as a choice option in different dimensions, e.g., when considering different objectives, or when enforcement is in place (see, e.g., bus bridging based on a disturbance).

One important application of information management lies in its ability to use more or less modern technology (machine learning, data-driven analysis, data science, etc.) to analyze and predict traffic patterns of public transport users. This could be based on smart card data, automated vehicle monitoring, real-time control data and alike.

Information provision towards the public is of utmost importance to attract possible customers towards using public transport. Especially in case of disturbances, customers need to be informed about implications of the disturbances. This can be done by means of collective information as well as individual information as indicated in the next paragraph. On the other hand, the provision of information can also bear disturbances in itself. If passengers are not or not sufficiently provided with action- or purpose-oriented knowledge, this may be seen as some sort of disturbance, too. A visualization of related issues by means of an appropriate 3-layer model (Wollnik 1988; Voß and Gutenschwager 2001) is provided in Table 2.

Coping with disturbances based on efficient information management is in the focus of Jevinger and Persson (2020, 2019). In a specific project related to disturbance management and information availability in public transport, the authors provide an outline of a prototypical information management solution focusing on a specific case in Sweden. However, the lessons learned are quite generic in pointing out which type of information each passenger might need in case of a disturbance. Marrying this with older ideas from, e.g. Daduna and Voß (1996), this encompasses different types of information flows like collective ones, where groups of passengers are informed about certain events or issues altogether, while individualized information provision addresses detailed needs of specific customers. Another source with the focus of real-time information in case of disturbances is Bruglieri et al. (2015). Information management-oriented case studies for Zurich, Switzerland, using agent-based simulation are Leng and Corman (2020) and Rahimi Siegrist and Corman (2021). A wealth of data is also provided through smart cards (Luo et al. 2019). Especially in rural areas with thinned-out transport structures, information needs become even more important in case of disruptions as clearly indicated by Papangelis et al. (2016).

Extending these ideas even leads to marketing issues and hidden disturbances; see, e.g., Echeverri and Skålén (2011). Co-destruction refers to the problem that objective information is often not available so that actions from one side (e.g., a bus driver closing the doors of a bus) can be misinterpreted by another side (the customer feeling uneasy about the door of the bus being closed) leading to collaborative diminishment of value by both actors. That is, one (the customer) sees a personal disturbance while the other one (the bus driver) sees the possible delay propagation without being able to explain to the customer that and why they have to wait. Social media use in case of disruptions is a different issue, well documented and surveyed in Pender et al. (2014b). Subjective user opinions of mobility networks can be used to judge upon the satisfaction level of users of mass transit systems, especially in the case of disturbances (Haghighi et al. 2018; Kokkinogenis et al. 2015).

Finally, note that information management implies rolling stock and public transport vehicles to become, in a sense, running information systems. If so, cyber security grows as a new threat and issue of disturbance; see, e.g., Schmittner et al. (2019).

### Network connectivity

Network connectivity has a major impact on measures such as robustness in transportation in general and especially in public transport. Nevertheless, it seems to be less understood in the latter domain. Therefore, we exemplify it in more detail. Impact can be deduced on all planning levels (e.g. strategic when to build a new line to increase the robustness of a transport system; e.g. operational when applying bus bridging after a subway station closure).

As mentioned before, robustness or fault tolerance can have different dimensions and different stakeholders that may be influenced by related measures. For instance, Liao and van Wee (2017) investigates a set of accessibility measures based on the number of travel options to express the robustness of a transport system, something that goes in line with classical facility location-related problems. Given an origin-destination (OD) pair, one may check for the connectivity between this pair of nodes in different ways. For instance, one can count the number of travel options connecting this OD pair. Given different modes of travel, e.g., one may resort to another mode if the former is no longer available. To make things more versatile, one might even change origin and/or destination, e.g., if one attempts to visit a shop of a certain brand or chain so that there is some sort of flexibility once a disturbance arises.

Usually, conditions of network serviceability are based on *connectivity*. That is, loosing connectivity is a major disturbance greatly influencing passengers. In this sense, different graph-theoretic concepts may be defined to measure connectivity (see, e.g., Derrible and Kennedy (2009) for some general exposition and Candelieri et al. (2019) for some case-study calculations) possibly indicating which networks might be more resilient or vulnerable than others. One should also bear in mind that transportation network representations in terms of graphs reveal some sort of special structure. Usually, for instance, a new line is appended towards an existing bus or train network with many nodes having a degree of 2 while at least one node connects to a somewhat important node of an existing network.

Actually, common sense tells us that in public transportation we have quite a few more or less important nodes. This can be a *central station* that connects to more lines than other stations or it can be a *hub* that accounts for incoming and outgoing traffic while allowing for transfer. Driven by the question on how to localize one or more most important node(s) in a network, various graph-theoretical measures may be defined and used (not necessarily invented for or used in the area of public transport).

Let us recall some graph-theoretical notation,^7^ i.e., we assume a given graph $G = (V,A)$ with node set *V* and arc set *A* where *A* is supposed to be a set of directed arcs. In case of undirected edges, we denote $G = (V,E)$ with edge set *E*. We assume *n* to be the (finite) number of nodes and *m* the number of arcs or edges, respectively. *d*(*i*) denotes the degree of node $i \in V$. The adjacency matrix $A_G$ of the graph *G* is an $n \times n$ matrix used to represent whether pairs of vertices are adjacent in the graph. Assuming a finite graph without multiple edges between pairs of nodes, $A_G$ is a (0,1)-matrix with zeros in its diagonal and a value of 1 indicating that an edge or arc exists, 0 otherwise. For undirected graphs the adjacency matrix is symmetric. Moreover, the eigenvalues are real numbers and the set of eigenvalues, let us denote them by $\lambda _{1}(G), \lambda _{2}(G), \ldots ,\lambda _{n}(G)$, are called the *spectrum* of *G*. The largest eigenvalue of the adjacency matrix denotes the spectral radius of *G* and is denoted by $\rho (G)$. Given two nodes *j* and *k*, we denote by $SP_{(j,k)}$ the number of distinct shortest paths that connect these nodes and by $SP_{(j,k)}(i)$ we account for the number of those paths that include node *i*. The following list gives a few measures as they may be used in public transport.

- Degree centrality $$D_{C}(i) = d(i)$$
  $D_{C}(i)$ denotes the degree centrality of node $i \in V$. As a simple measure we use the degree of a node with the tendency that a node is more vulnerable the larger its degree is. The vulnerability of a node may be seen regarding the node itself as well as the influence of the removal of a node on the performance of a transportation network. In the first case, the vulnerability of a node increases the more complex its structure is. Consider, e.g., a (railway) station with many incoming and outgoing edges and related crossings of lines, then there is a higher chance for a disturbance (e.g. based on an accident) compared to a node with small degree centrality. For further reading see Sugishita and Asakura (2021) and the references therein. According to Du et al. (2014), a network node may also be seen as vulnerable if the loss (or substantial degradation) of a number of links significantly diminishes its accessibility.
- Betweenness centrality $$B_{C}(i) = \frac{1}{n (n-1)} \sum _{i \ne j, i \ne k}\frac{SP_{(j,k)}(i)}{SP_{(j,k)}}$$
  $B_{C}(i)$ denotes the number of times a node acts as a connection along a shortest path between two other nodes again with the tendency that a node is more vulnerable the larger its betweenness centrality is. In case of betweenness centrality we may argue in a similar way regarding the vulnerability of a node and the vulnerability of a network as done for degree centrality. For a case study using the Shanghai metro network, we refer to Sun and Guan (2016).
- Spectra of graphs
  The spectral radius of graphs as well as the spectra of graphs (especially using the second eigenvalue of an adjacency matrix) may also be used as important measures characterizing the significance of certain nodes and edges in public transport networks, although this has largely been neglected outside the field of graph theory; for exceptions regarding public transport and transportation networks in general see Maas (1987), Candelieri et al. (2019) and Bell et al. (2017). Due to the lack of further references regarding public transport, we acknowledge the closeness to telecommunication networks allowing to refer to Çetinkaya et al. (2015).

Different types of disruptions may imply different measures for proper judgement. Moreover, different types of networks are more vulnerable than others. For instance, a network with a specific node with a large degree centrality and a large betweenness centrality is more manipulable by means of a terrorist attack in that node than in others. Looking at a specific node by itself, there seems to be a higher vulnerability, e.g., regarding possible delay propagation based on traffic jams and disturbances around and even inside the node.

The general settings on network connectivity and related measures can be found in Bell et al. (2017), Mishra et al. (2012), Kindlmann and Burel (2008) (without cross-referencing in these references). While Dimitrov and Ceder (2016) do not provide a thorough review of fundamental works in graph theory, they help to understand the idea of scale-free and small-world networks in public transport. Using a property for description, scale-free networks can be characterised such that some nodes have a very large number of connections to other nodes (central station, hubs), whereas most other nodes have a very small number of connections (like many stations along a line connecting a hub or the central station with some outside city or a terminus in the boondocks).

*Redundancy importance* is introduced by Jenelius (2010) in considering two measures based on traffic flow and disruption impacts (operationalized as travel delay) given that the measure is not related to a network under normal conditions but assuming its importance in case of being used under disruption. That is, such a measure aims at indicating the importance of a connection under the assumption that others are disrupted. A slight extension and an application of this exposition can be found in Jenelius and Cats (2015). Using various measures described in this subsection can support the investigation of the vulnerability of public transport networks as, e.g., in Mouronte-López (2021). Considering multi-level networks (e.g., those emanating from different means of transport) can be found in Baggag et al. (2018). Based on a comparison for major cities with a multitude of modes (Chicago and New York, USA, London, UK, and Paris, France), the authors claim Paris to be more robust than the others in terms of coverage degradation after removing a small fraction of edges.

## Classification: from prevention to reaction

Taking actions regarding disturbances can be classified along the timeline to especially distinguish what happens before and after a disturbance occurs. In this section, this is elaborated in general terms while Section 5 provides specific problem settings in the spirit of public transport planning and operations.

**Fig. 4:**
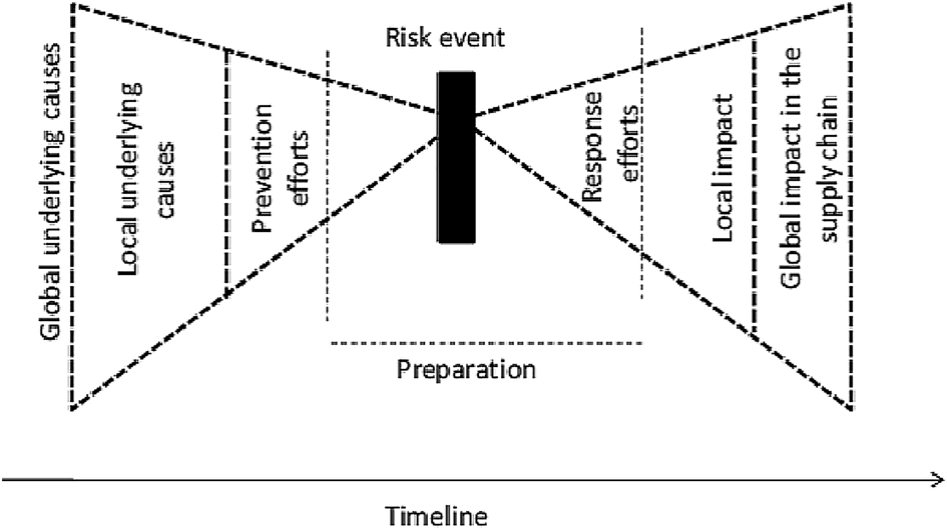
Butterfly depiction of supply chain risk (Sodhi and Tang 2012, p. 16)

Considering the timeline in general terms, one may distinguish *prevention* and *reaction*. Occasionally, especially in supply chain risk management, this comes along with a “butterfly” depiction as a helpful way to identify events; see Fig. 4 (Sodhi and Tang 2012; Dadfar et al. 2012). The left-hand wing represents underlying global and local causes that could lead to a *risk event* (another word for disturbance) as well as prevention (i.e. proactive) efforts that are undertaken before the occurrence of the event, while the right-hand wing delineates the local and global impact of the risk event and response efforts (i.e. reaction) made after the event has occurred.^8^ Moreover, this model can be modified to depict causes in the upstream supply chain (left wing) leading to effects downstream in the supply chain (right wing). The abscissa reflects not only the time, i.e. for prevention and response efforts as well as preparation to the response, but also the relative location of the causes, the event and the impact in the supply chain. Sometimes risk events are anticipated and the preparation for response can start prior to the event. For instance, a possible labor dispute at a local rail company could cause a switch to alternative transportation modes. Organizing reserve shifts for possible events to happen may be a different example. Later we shall use the notion of *anticipation* in this respect.

**Fig. 5:**
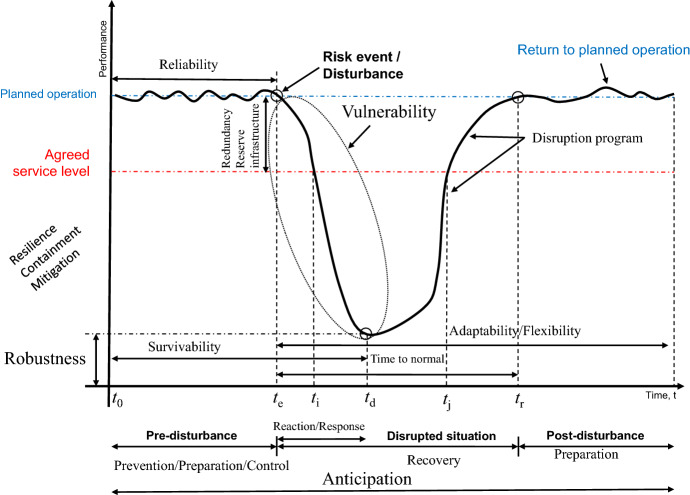
Schematic presentation of the performance of a resilient system closely related to Wan et al. (2018, p. 491); a similar figure can be found in Taylor (2017, p. 45)

A specification of such a figure in the above spirit is provided in Fig. 5. The figure shows a hypothetical system performance under normal conditions and in face of a disruption. Up to that point the operations run as planned and the time until such a point is occasionally called survivability in supply chain management. The timeline indicates a risk event or disturbance at time $t_{e}$. Given certain service level agreements, reserve infrastructure and redundancy may support the case and keep the system and its performance beyond agreed service levels. Different means of resilience from the literature, as understood by Wan et al. (2018), are presented. Performance can be conceived as the services offered and to which extent they are functioning. Before a disruption occurs, the system operates as planned. At a certain time a disruption is encountered (for a specific description of the detailed functions and time points see the original source) degrading performance. The response effort is supposed to imply recovery until the system is back to the original state. Adaptability and flexibility as in supply chain management may also be related to degrees of freedom.

### Prevention

Prevention can mean maintenance and repair (pre-planned disruptions; see Section 5.8), it can mean extra buffer times etc. Actions to cope with prevention issues include many fields of action like improved schedule synchronization (see, e.g., Nesheli and Ceder 2015), the investment in spare capacity, buffers and alike. Some general statements regarding reliability and related measures can be found in Nicholson et al. (2003). Some specific issues with applications to ferry services in HongKong are provided in An and Lo (2014). In Zieger et al. (2018) a Monte-Carlo simulation is used to show that line capacity can vary by as much as 17% as a function of the underlying buffer time statistics. Even more so, if it is known that trains are extremely crowded, one may take some preventive measures in determining stopping patterns based on travel times as well as (expected) congestion rates of trains. In Yamauchi et al. (2017, 2021) the authors optimize stopping patterns for train-based traffic in Tokyo, Japan, utilizing a Wardrop equilibrium model regarding passenger flows as well as a local search approach to optimize stopping patterns.

A *contingency plan* is a plan made for the case that something happens which is different from the usual (expected) plan. This is something that can be done proactively. A literature review from Parbo et al. (2016) includes quite a few qualitative statements especially regarding the valuation of contingency plans by passengers. As a handwaving issue, many things do not come as a surprise. For instance, if bad weather is announced (snow, storm), preventive measures may be taken. Of course this requires awareness about possible correlations between potential disturbances and related events; see, e.g., Ling et al. (2018).

Among proactive approaches we see the advent of disruption programs (DRPs). DRPs are sets of pre-defined dispatching measures in case of certain (infrastructural) disruptions (Chu and Oetting 2013). The goal is twofold. First, it is aimed to ensure somewhat stable operations during a disrupted situation. This could incorporate the use of different means of transportation (see, e.g., short turning and bus bridging in Sects. 5.5 and 5.6). Second, they should envisage the movement towards formerly planned operations once the disruption is overcome. As DRPs are prepared upfront, they are faster to implement and easier to communicate than ad-hoc dispatching measures.

On a different scale, proactive approaches also include the investment into taking certain measures to avoid disruptions or to be able to recover quickly (or, e.g., smoothly) from them. These investments may depend on which type of disruptions should be possibly covered. Thinking of terrorist attacks, they could include infrastructure provision for permanent security measures like luggage screening similar as it happens at airports. That is, they should be different than, e.g., if they just concern possible redundancy in case of lift or escalator outage. An example for transportation-oriented studies in this respect is Cox et al. (2011).

Even if one thinks about evacuation plans in public transport, preventive actions can be taken. That is, while we mostly discuss disturbances in public transport, we may change roles and ask to which extent public transport can help to support reaction after major societal or similar disturbances in society. In TRB (2008) and National Academies of Sciences (2013), the development of a guide on the role of not just public transport but transportation as a whole in all-hazard emergency evacuations is described. Actually, this also results in requirements to be fulfilled by today’s public transport companies such as the following: “Transit should have the capability for real-time interoperable communications (both voice and data), be part of the decision-making team for emergency operations, develop effective ways of communicating with transit passengers both in advance of and during an emergency, ...” (TRB 2008, p. 128). Important characteristics of a public transit system to allow judging on its capability to support, include the following: Size of the system and coverage of its service area, the modal mix and flexibility of the system and its type of service (e.g. directly provided or contracted out) as well as the condition and capacity of the system.

### Reaction

One of the classifications of disturbances distinguishes minor and major disturbances; another distinguishes disturbances with very small probability and those with a higher one. A reaction itself can have many dimensions depending on the type, length etc. of the disturbance as indicated in Table 1 above. Based on this, we also find many different characterizations within the reaction category. These include emergency management, containment, recovery and delay management.

*Emergency management* goes beyond disturbances in public transport and refers to the management and organization of resources and responsibilities for dealing with any aspects of emergencies. While this already starts with preparedness or preparation (see Fig. 4), most of it relates to reaction incorporating response, mitigation, and recovery. The foremost concern is to reduce the harmful effects of all hazards, including major disturbances, disasters and alike. An important characterization is that immediate response and immediate measures need to be taken (like in case of a terrorist attack). A common distinction also distinguishes between disaster and emergency management where the latter is often related to issues aiming to prevent a disturbance from turning into a disaster. With this, response efforts include *containment* which means to keep something happening within limits (like avoiding that an emergency turns into a disaster). Similarly, *mitigation strategies* are strategies intended to reduce the impact or effects of a disturbance.

Once immediate emergency actions have been taken, one resorts to recovery. Though, the classification of what reaction and/or recovery could mean also depends on the eye of the beholder. Various means of reaction or recovery may be distinguished by means of who is going to be in the focus or who is going to take action. While thinking usually goes in the direction of a transport provider taking actions, it may also relate to the passenger taking actions (like seeking immediate alternatives towards continuing a disturbed journey up to and beyond changing loyalty patterns regarding public transport (providers)). Short-term actions include immediate reactions like waiting, mode change, etc. while long-term actions may even incorporate a change in modal split. To a large extent this area touches behavioral models up to policy matters. Examples include Monchambert and de Palma (2014), Saxena et al. (2019) and Adelé et al. (2019). Topics considered include value of time, value of punctuality, willingness to pay, etc. Often questionnaire-based or stated preference-based studies support research in this area, although many of them lack appropriate lessons learned. Most influential is the work of Eboli and Mazzulla (2007). While they are mostly cited due to their general exposition regarding structural equation modeling in public transport, their considerations related to our paper refer to reliability concerning buses that are on time. This is a major issue of many studies including, e.g., Mouwen (2015) and Rahimi et al. (2019). Seldom well-performed questionnaire-based research puts the exposition into relation with preferences regarding the use of scarce resources. For instance, investigating perceptions of risk and safety and putting that into perspective regarding priorities for improvement would be an example; see, e.g., Thomas et al. (2006).

While delay management and recovery as well as mitigation strategies seem closely related, the references are to a large extent distinct (cf. Sect. 5 with some subsections like Sect. 5.4). These topics are treated, e.g., in Pender et al. (2013), Malandri et al. (2018) and Zeng et al. (2012). Among these papers, Pender et al. (2013) provides a survey based on semi-structured interviews of 71 transit companies/agencies.

In a similar spirit one may also think about keeping reserve infrastructures, (e.g., buses) especially for coping with disturbances in peak hours. This can be a general issue but may also be used for *bus bridging* (see Sect. 5.6). This term is used to describe the situation of substituting services in case of a disturbance making rail-based transit unavailable for some time in a way where affected stations are “bridged” using bus services.

Another idea amenable for reaction purposes as well as anticipation would be to (pre-)calculate templates of similar solutions that might come into effect in case they are needed. While this idea has not yet been investigated with a focus on disturbances and robustness in public transport, it is already established with a different focus in Borndörfer et al. (2013) where similar duty schedules are determined for similar days of operation. Duty templates can possibly be used to minimize changes to the plan when rescheduling becomes necessary.

*Real-time control* can be used as a means to detect and analyze disturbances beyond resolving them (Dridi et al. 2005). With that it may have an important impact on possible reactions. A survey on related literature until 2015 can be found in Ibarra-Rojas et al. (2015). One of the things possibly discovered may be bus bunching (as indicated in Sect. 5.7 below).

Once problems have been resolved, the reaction domain still needs to incorporate the aftermath of the disturbance to let all involved stakeholders learn based on the event. This kind of analysis resorts to many different directions like data-driven approaches, machine learning and forecasting. To exemplify, we refer to a simple analysis of delay and punctuality data for a specific area in Europe (Økland and Olsson 2021). Data from automated vehicle monitoring, smart cards and real-time control can be analyzed in the same spirit.

## Solution approaches

Planning and operations usually go hand in hand with operations research (OR) methods. From an OR perspective, solution approaches usually attempt to solve problems exactly or heuristically.^9^ The first works in many cases by means of setting up a mathematical programming formulation like an integer programming or a mixed-integer programming (MIP) model and using related standard solvers like CPLEX or Gurobi.^10^ However, especially due to the complexity of the underlying problems, related models can often not be solved in time limits deemed practical. Therefore, one also resorts to heuristics and metaheuristics.

As a general observation, we may state that this field relies a lot on modeling and solving problems by means of relating them to well-known combinatorial optimization problems, such as the set partitioning problem and the set covering problem. Given a set of items *S*, the *set partitioning problem* aims to determine how the items from *S* can be partitioned into two or more smaller subsets. In this problem setting partitioning means that all items from *S* must be contained in exactly one of the subsets. With a slightly different view, if one would know the number and types of subsets, this may also be seen as an *assignment problem* as the items need to be assigned to the subsets. In the *set covering problem* we are again given a set of items *S* as well as another set of subsets of *S*. The question is to find a collection of these subsets, e.g., a minimum number of them, so that the union of all the elements of the chosen collection of subsets includes all the elements of *S*. The set partitioning problem and the set covering problem are classical examples of $\mathcal {NP}$-hard or $\mathcal {NP}$-complete problems, depending on whether an optimization version or a decision version is considered. We may use these well-established problems as subproblems for a large variety of problem settings in public transport. For instance, given a set of duties and a set of drivers, the drivers have to *cover* the duties. Or, every trip has to be driven by one vehicle, etc. Therefore, many problems in our settings are formulated as (generalized) set partitioning and set covering problems and this is also reflected regarding the development of solution methods.

We start to single out one application (i.e. Abbink et al. 2005) as it striked the OR community regarding the success of those approaches in real-world crew scheduling settings. In addition, a general exposition on algorithmic aspects for railway disruption management including especially the set covering problem can be found in Kroon and Huisman (2011). Examples they are refering to include Potthoff et al. (2010) and Rezanova and Ryan (2010).

Before going into more detail regarding solution approaches, we should note that most problems tackled in this paper have a single objective while some of them are multi-objective in nature (with bi-objective acting as a special case). We start with a few general hints towards heuristic, metaheuristic as well as exact problem solving. Then, we are propagating robust optimization. The section closes with pointers towards a few other concepts.

### Heuristics and metaheuristics

In simple words, a *heuristic* is a more or less simple or even versatile rule of thumb or method for solving problems without guaranteeing optimality. Heuristics provide (simple) ideas to indicate which among several alternatives or choices for solving a problem seems best. *Greedy heuristics* are simple iterative methods with myopic behavior. Starting with a given feasible or infeasible solution, iteratively one out of a number of alternative choices is taken to fix or modify one or more variables. For heuristics we distinguish those for finding initial feasible solutions and those for improving them.

The next important concept to be considered is *local search* where given solutions are successively altered. Related transformations are characterized by neighborhoods which incorporate solutions obtained by iteratively moving from one solution to another (e.g. by changing the value(s) of some variable(s)). Large scale neighborhoods are to some extent going beyond simple neighborhood structures, eventually using some sort of destroy-and-repair mechanisms. This also resorts to the possibility of strategically oscillating between feasibility and infeasibility of the solutions. This also leads to the notion of *metaheuristics*. “A metaheuristic is an iterative master process that guides and modifies the operations of subordinate heuristics to efficiently produce high-quality solutions. It may manipulate a complete (or incomplete) single solution or a collection of solutions at each iteration. The subordinate heuristics may be high (or low) level procedures, or a simple local search, or just a construction method. The family of metaheuristics includes, but is not limited to, adaptive memory procedures, tabu search, ant systems, greedy randomized adaptive search, variable neighborhood search, evolutionary methods, genetic algorithms, scatter search, neural networks, simulated annealing, and their hybrids” (Voß et al. 1999, p. ix). For an in-depth survey of metaheuristics the reader is referred to Caserta and Voß (2009). Beyond metaheuristics we also have *matheuristics* which are optimization algorithms characterized by the interoperation or hybridization of metaheuristics and mathematical programming techniques (Maniezzo et al. 2009). An essential feature is the exploitation in some part of the algorithms of features derived from the mathematical model of the problems of interest, thus occasionally one also finds the notion of model-based heuristics.

Providing a survey on all types of heuristics and metaheuristics applied to robustness- and disturbance-related problems in public transport is beyond the scope of this paper. Literally, almost anything has been tried or may be tried and some pointers are/were given on the fly. Considering the problem settings from public transport planning and operations, it becomes obvious that, beyond heuristics and metaheuristics, there is an inherent notion of decomposition-based approaches (see, e.g., Desaulniers and Hickman 2007). That is, many of the problems presented have some sort of natural decomposition into subproblems (like the set partitioning problem). While some of them are easily solvable, others are still difficult. Examples include the discussion of the integration of different problems like in the integrated vehicle and crew scheduling problem. Here the decomposition into subproblems should naturally lead to the use of matheuristics incorporating mathematical programming approaches, e.g., for solving problems like the set partitioning or the set covering problem as subproblems. In that sense, a lot of research in our area is devoted to properly decomposing problems into subproblems amenable to efficient solvability.

It is in the ingenuity of the modeler and algorithm developer to provide proper ideas for decomposition as can be found in the development of many matheuristics. Ideas include, e.g., to heuristically fix some difficult variables while solving the resulting problem to optimality (or even heuristically) with related approaches. Let us deviate from classical review papers and exemplify by sketching some ideas and providing some food for thought. Consider, e.g., the concept of *Lagrangian heuristics* as it has been used successfully in the context of our paper by Cacchiani et al. (2012). Given a mathematical programming formulation of a problem, one may solve a relaxed problem (exactly or heuristically) by means of an iterative Lagrangian optimization scheme. If robustness parameters are introduced into the formulation, it might still work well and the way of advising subproblems may change by incorporating robustness in a stepwise fashion.

Another concept is to decompose the problem into parts by means of tentatively fixing variables and optimizing those that are not fixed. With respect to matheuristics this could go back to older ideas to involve the POPMUSIC approach (recently applied in Doi et al. (2018)) or even a heuristic Benders approach (Mesquita et al. 2013). In a metaheuristic fashion one may resort to older ideas of chunking or consistent chains. In the quest for robust solutions in vehicle scheduling this has been renamed as stable chains by Gintner et al. (2005). A given problem is decomposed into several simpler problems and solved many times. Overlapping parts of those solutions are then determined as “robust sequences” of trips. Extending common greedy heuristics, this is called fixed set search in a modified setting in Jovanovic et al. (2019).

### Exact methods

Exact methods for solving public transport problems under robustness and disturbance assumptions include about anything known from OR and other areas. Examples are decomposition approaches (including, e.g. Benders decomposition), branch and bound (including branch and price) as well as column generation, dynamic programming etc. A good linkage of these methods with public transport planning and operations is Desaulniers and Hickman (2007).

### Robust optimization

In this paper, among others, we depart from the classical approach to problem solving in public transport and study related problems in the context of a robust optimization framework as well as various measures considering disturbances. And, as related words are used in different ways and with different meaning, we aim at connecting different strands of literature. Mostly, researchers resort to two main classes of methods proposed in the literature to handle uncertainty: stochastic programming (offering great flexibility, but often leading to models too large in size to be handled efficiently), and robust optimization (whose models are easier to solve but sometimes lead to very conservative solutions of little practical use).

Let us start by stating that the importance of *robust optimization* in management science has long been recognized. In their seminal paper “Making the Case for Robust Optimization” (Bai et al. 1997), the authors claim that it is important to embed uncertainty (let us use this term for some sort of disturbance or some sort of interference) into the decision-making process. They state that missing out on incorporating uncertainty in decision making may have “very expensive, even disastrous consequences if the anticipated situation is not realized” and it is concluded that finding those solutions which are not too sensitive to any specific realization of uncertainty is most important. In that sense, again, *robustness of a system* can be seen as the ability to keep up its functionality under conditions that deviate from normal. Stating in different words, the exact value, e.g., of the input data is not known in advance and can be affected by uncertainty. Robust optimization aims at finding solutions which are not too sensitive towards acknowledging uncertainty. An overview for different methods about robust optimization, both in theory and applications, can be found in Gabrel et al. (2014). A common understanding is that one may assume the worst case in all or just some dimensions.

In Bai et al. (1997) it is shown that, at least with respect to the considered utility functions, the nonlinear programs arising from the robust optimization formulations are not much more difficult than their linear counterparts. In case of capacitated facility location, it is even possible to develop generic approaches that apply equally well for single-source, multiple-source, as well as deterministic and stochastic versions (Caserta and Voß 2020). In public transport we rarely see the formulation of robust versions of otherwise well-known optimization problems. Examples may be problems where demand is uncertain, like in Qi et al. (2018), the integrated vehicle and crew scheduling problem with days off patterns in Ge et al. (2022), the robust version of the periodic event scheduling problem in Goerigk (2015), and alike.

In a similar spirit, the idea of *light robustness* is discussed in Fischetti and Monaci (2009) which can be seen as a heuristic version of robust optimization. For the arising optimization problems, the authors define a robust solution as a possibly suboptimal solution whose feasibility and cost is not affected heavily by the change of certain input coefficients. Light robustness couples robust optimization with a simplified two-stage stochastic programming approach, and has a number of important advantages in terms of flexibility and ease of use. In particular, experiments on both random and real-world problems show that light robustness is sometimes able to produce solutions whose quality is comparable to those obtained through stochastic programming or robust models, though it requires less effort in terms of model formulation and solution time. The application setting in Fischetti and Monaci (2009) is train timetabling. We should note that a mathematical exposition showing the impact of light robustness and some idea to generalize the concept can be found in Schöbel (2014).

Using the idea to replace an uncertain optimization problem by a deterministic version may also lead to something called *recoverable robustness*. Simply spoken, it can be seen as a method to cope with uncertainty combining robust optimization and a two-phase stochastic programming approach, where it is important to be able to make solutions feasible after the first stage. The concept together with some timetabling applications is described in Liebchen et al. (2009).

### Miscellaneous

*Model-predictive control* (MPC) is an online optimization-based control approach that optimizes a given problem (characterized by an objective subject to a given set of constraints); see, e.g., Heilig et al. (2015) and Nabais et al. (2012). The idea of using MPC in our context is to construct models that describe the behaviour of a transport network and perform predictions over a certain time horizon based on continuously measuring the current state of the network or system by means of, e.g., sensor technology. Given those predictions, an MPC control agent determines at discrete control time events or control steps the actions to be chosen in order to obtain the best performance regarding, e.g., in terms of headway adherence, by solving respective optimization problems considering desired goals, existing constraints, environmental factors, and existing forecast information. The solution can be implemented by using actuators or based on information exchange among involved actors. Examples for using MPC in public transport within our context can be found in Caimi et al. (2012), Andres and Nair (2017) and Blenkers (2015). Moreover, various machine learning techniques may be used for prediction using a wealth of data sources. That is, data-driven approaches incorporating machine learning may support the case of anticipating situations before, during, and after some disturbance.

An important class of approaches is coming from (discrete-event) *simulation*. Discrete event simulation deals with the modeling of dynamic systems. The state of a dynamic system is described by means of time-dependent state variables which change their state at certain (discrete) points in time. That is, in discrete-event simulation one models the operations or behavior of a system as a (discrete) sequence of events in time. Simulation-based optimization then hybridizes or integrates optimization techniques with/into simulation analysis. An example for limited-stop bus service with vehicle overtaking is provided in Wu et al. (2019). The train rescheduling problem being treated with simulation-based optimization can be found in Shakibayifar et al. (2018), and in Hassannayebi et al. (2016) a line blockage disruption is investigated where the disruption model combines short-turning and station-skipping control strategies. A more general survey incorporating different control strategies is presented in Gkiotsalitis and Cats (2021). They advise a combination of control measures, passenger-oriented decision making, coordinated network control, bus deployment and disturbance management.

A special class of simulation models are *Petri nets*. They offer a mathematically founded graphical notation for stepwise processes that include choice, iteration, and concurrent execution. Based on Petri-net simulations, one may investigate control strategies that either address an occurring disturbance immediately or, alternatively, modify sojourn times while being on track and also account for accumulated delays. Out of a group of many very similar papers by the same group of authors partially even without cross-referencing, we mention Mhalla and Gaied (2018) and Gaied et al. (2019). An example of using Petri nets regarding BRT and bus station design is given in Gonzalez-Lopez et al. (2017).

Robustness and disturbances in public transport may call for completely different types of solution approaches, where not all of them are solution approaches in an OR sense (like what we described as robust optimization or light robustness in Sect. 4.3). That is, more related to a transport economics focus one may also think in terms of governance structures and policy development. Related to robustness, one of the unanswered issues in this paper relates to the question of ownership and disaggregation, a classical policy issue in transportation. For instance, the question whether regulation is going to separate the ownership of the infrastructure and the services run on this infrastructure may have a major impact on robustness and disturbances and how to cope with them. Moreover, the legal constraints bound in concession contracts being about how to compensate in case of disturbances is another interesting issue. However, these are beyond the focus of this paper. For an entry into this strand of literature see, e.g., Karl (2018) and European Parliament (2011). In addition, we refer to Hensher et al. (2016) regarding possible transition costs, perceived or real, that may be relevant when evaluating concessions, concession transitions as well as performing competitive tender evaluation.

## Problem settings

After having specified some methodology as well as the butterfly depiction and the idea of prevention and reaction or the idea of going from being proactive towards being responsive, we clarify specific problems belonging to one or the other idea or concept. That is, we classify along the lines just developed and provide a few examples (with forward pointers as we specify in more detail below):

- *Proactive approaches*

- Example: Build an evacuation plan
- Example: Extend capacity; this can be devoted towards network design (see Sect. 5.1)
- Example: Add buffer times to encounter primary delays (see Sect. 5.2) and propagated delays (see Sect. 5.3)
- Example: Maintenance and repair (see Sect. 5.8)

- *Reactive approaches*

- Example: Delay management (see Sect. 5.4)
- Example: Short turning (see Sect. 5.5)
- Example: Bus bridging (see Sect. 5.6)

- *Anticipation*

- Example: Ask for spare capacity
- Example: Build reserve shifts (see Sect. 5.3)
- Example: Forecasting and prediction (see the hints in Sect. 4.4)

Note that problem settings in most papers are related to planning *for* the public transport service provider and for the customer. Changing views could also include planning *of* the customer (see Sect. 5.9).

### Network design

Network design is closely related to problem settings like line planning, station design, etc. Thinking of strategic planning with regards to a new transit line, we typically see a sequential or hierarchical planning process with network design being first. Integrating the first two processes, i.e. transit route network design and determining frequencies, is often called the transit network design and frequency setting problem.

An example of using robustness in connection with classical notions of transit network design is Yao et al. (2014). Their optimization model takes into account stochastic travel times while satisfying passenger demand and reliable transit service. In Cats and Jenelius (2015) a methodology for assessing the value of capacity increase for network robustness is discussed and exemplified for network design in the context of Stockholm, Sweden. On a strategic level, increasing capacity usually goes along with improved robustness (Cats 2016). For exceptions we refer, e.g., to the well-known Braess paradox (Braess 1968; Jenelius and Cats 2015); here, simply spoken, added capacity can actually worsen the traffic flow.

An online predictive optimization framework for the transit network design and frequency setting problem is presented in Peled et al. (2019). The framework aims to combine demand prediction and supply optimization (regarding the offering of transport services) to periodically redesign the service routes according to the observed demand within the most recent history.

In different settings, it is also encountered that the (bus or train) station design has an influence on possible disturbances (Voß et al. 2020). At stations, capacity limitations can be a major reason for delays and delay propagation. Bus station design with Petri nets is considered in Gonzalez-Lopez et al. (2017). Supporting the robustness in relation to a station and avoiding potential conflicts can be accomplished by maximizing the spread of the trains (Dewilde et al. 2013). Literally this means optimizing the routing of trains to the available platforms. A sensitivity analysis could also imply some beneficial changes in timetabling, an issue that has not yet been investigated in conclusion. Moreover, the interplay between the spread of trains and schedule synchronization is not yet fully explored as we are encountering conflicting objectives.

Network design also relates to determining critical infrastructure. For instance, using ideas from Sect. 2.6 may lead to measures of resilience, e.g., regarding critical nodes within public transport networks (Zhang and Ng 2021). Among others, this leads to issues of redundancy allocation (Caserta and Voß 2015), which have, so far, not comprehensively been studied in public transport planning and operations.

A survey on selected literature focusing on network design can be found in the appendix (see Table 3).

### Timetabling

A survey on papers regarding robustness in railway planning by Lusby et al. (2018) concludes that most of these works are devoted towards timetabling. Moreover, they discuss various ideas for measuring robustness as it can be found in literature. Regarding timetabling, practical considerations can classify disturbances in different ways. For instance, in case of demand fluctuation due to a major sports event this may be classified as an operational uncertainty with a separate timetable or as a disturbance. For a comprehensive survey on methods for the (nominal) train timetabling problem as well as the robust train timetabling problem we refer to Cacchiani and Toth (2012, 2018).

In Solinen et al. (2017) the authors focus on constructing robust timetables that aim to allow trains to recover from delays as well as preventing delays from propagating. Their approach uses an indicator called robustness in critical points (RCP) as well as a method to possibly improve the RCP. A case study is presented where an initial timetable and a timetable with increased RCP values are evaluated.

In Qi et al. (2018) an integrated train timetabling and stop planning problem (TTSP) is defined. Given a set of trains, the idea is to determine for each train a subset of available stations that the train is bound to serve. The latter is called a stop plan. Moreover, given passenger demands for a set of OD relations, the timetable and the stop plan are to be determined. Assuming passenger demand being uncertain, the problem is extended towards the Robust TTSP in the spirit of what we proposed in Sect. 2.3. The authors use an integer linear programming (ILP) model for this problem based on the idea of applying the concept of light robustness. A case study is provided for the Wuhan-Guangzhou (China) high-speed railway corridor under different demand scenarios.

Another important issue also belonging to the reaction realm relates to the impact of delays beyond delay management; see, e.g., Friedrich et al. (2018). The authors compare timetables that have been optimized with different strategies to increase robustness by inserting buffer times. Random delays are investigated in simulations based on historical observations. A major concern relates to whether fixed or variable buffer times should be added. In Jovanović et al. (2017) fixed buffer times are allocated to protect events according to their priorities. A simple idea is to allocate buffer times by formulating a resource allocation problem as a knapsack problem. Here buffer times may be considered as items with a value according to given priorities coming from company-related criteria while the weight is given as the time duration. A case study from Sweden is reported.

The train rescheduling problem concerns the real-time resolution of conflicts arising during train operations. Given a nominal timetable for a set of trains as well as some disturbances, the goal is to determine a set of actions to be implemented to resolve the resulting conflicts. This includes the avoidance of train collisions or headway violations as well as the restoration of the system. References on this problem include, e.g., Bettinelli et al. (2017), Shakibayifar et al. (2018), Corman et al. (2012), Zhan et al. (2016) and Yin et al. (2016). The train rescheduling problem may also be classified as being part of recovery and mitigation strategies. An important feature is the requirement of real-time compatibility as possible conflicts like collision avoidance need to be resolved immediately. Therefore, very fast algorithms are needed, implying to resort to simple greedy heuristics and simulation approaches. That is, the train rescheduling problem is closely related to recovery models and algorithms for real-time disruption management. A survey can be found in Cacchiani et al. (2014). A multi-objective approach focusing on different stakeholders can be found in Binder et al. (2017).

An interesting option in timetabling is to vary the number of stops to call at. This could mean a variation in the number of stops and especially to skip some stops. This has an impact on the possible demand to satisfy as well as the track capacity; see Jiang et al. (2017) and Jamili and Pourseyed Aghaee (2015). This idea can also be applied in the context of bus bunching (Sect. 5.7).

A survey table with references regarding disturbances and timetabling is given in the appendix (Table 4).

### Vehicle and crew scheduling

While the previous subsection is devoted to timetabling, we now incorporate vehicles and crews. This encompasses scheduling and rostering.

An important reactive measure after a disturbance is *rescheduling* (or rerouting). First of all, one may use the same models and methods to solve a new problem instance arising after a disturbance. As an example, assume that a node, a link or a sequence of links in a rail-based system is no longer available. Then, beyond the notions of bus bridging or short turning (see Sects. 5.6 and 5.5), repeating and rerunning existing approaches on the modified network might be an option.

The work of Cacchiani et al. (2014) presents an overview of recovery models and algorithms for real-time railway disturbance and disruption management. A specific recovery model incorporating holding as well as speeding is proposed in Wu et al. (2018). Also Fang et al. (2015) provide a survey on rescheduling issues. Cadarso and Marín (2014) propose an integrated model for timetable and rolling stock rescheduling in order to minimize the recovery time, the passenger inconvenience and the incurred system costs. While this is closely related to timetabling, one may also think of a separate category.

In Rezanova and Ryan (2010) the authors consider a train driver recovery problem that needs to be solved immediately after the occurrence of a major disruption in the daily railway operations. This recovery problem is formulated as a set partitioning problem after a modeling exercise defining nested disruption neighborhoods. First, a small set of drivers and train tasks directly affected by the disruption is defined, the model formulated and possibly solved. If a feasible solution is found, the procedure stops. Otherwise, the neighborhood is extended by adding more drivers or increasing the recovery time period. This is consecutively repeated and married with the solution of linear programming relaxations of the related model. In Potthoff et al. (2010) the authors utilize set covering constraints for the problem of rescheduling crews at the time of a disruption considering necessary changes in the timetable and the rolling stock schedule. The problem under consideration is called operational crew rescheduling problem. Robust vehicle scheduling, scheduling electric vehicles and environment-friendly vehicle scheduling are topics within van Kooten Niekerk (2018) that are tackled by means of set covering and set partitioning problems.

The crew rescheduling problem with retiming is considered, e.g., in Veelenturf et al. (2012). They extend the crew rescheduling problem by the possibility to slightly delay the departure of some trains to allow more flexibility in the crew scheduling process. In a sense this relates to a sensitivity analysis of schedules in, e.g., modifying the departure time of some trains to allow more flexibility in the crew scheduling process. Papers considering automatic rescheduling and interactions with regular railway operations include Corman and Quaglietta (2015) and Fan et al. (2012)).

In Veelenturf et al. (2016b) the idea of quasi-robustness is applied to crew (re-)scheduling. Given a partial plan, the idea is to generate completions for this plan (e.g. regarding drivers) which is simply assuming that feasibility can be achieved. For a disturbed system this can be done while minimizing rescheduling costs. If not all of these completions are robust but only some of them, the authors call that quasi-robust rather than robust.

An interesting approach is one that defines a bi-level rescheduling algorithm using a MIP model combining macroscopic and microscopic modelling elements. The idea of Cavone et al. (2019) is to formulate this model to obtain a feasible rescheduled timetable incorporating safety constraints as well as capacity and ordering constraints for the disrupted stations. Numerical results are presented for rescheduling Dutch railway traffic in case of a full blockade between two consecutive stations.

If uncertainty about the time of an event is considered, this may be incorporated into planning processes by means of buffer times (or extra capacity). Different from delay management as discussed in Sect. 5.4, public transport vehicles may be trapped in traffic jams with implications for subsequent trips or even the usability of vehicles (or adherence of the legal constraints on the driver’s working hours). To specify, consider battery-driven buses. Even for recent generations of these buses this may become a challenge regarding their range and buses might need to get back to a depot for battery change or charging earlier than expected. In the same spirit, as encountered with demand-responsive transport as well as various service providers in an MaaS setting (eventually even using autonomous vehicles), the number of available vehicles may change according to outside circumstances. This may include weather conditions, driver capabilities (with a varying battery utilization based on temperature, driver behavior, recuperation implementation) and alike. Selected reading includes Vepsäläinen et al. (2018, 2019). Also with many sharing concepts, the number of available vehicles may change over time and is dynamic. In Tang et al. (2019) this topic is investigated by means of robustness in static and dynamic (vehicle) scheduling models. The static model introduces a buffer-distance strategy to tackle the adverse impacts caused by trip-time stochasticity. From a modeling perspective, to achieve this, the authors propose to define a capacity constraint in which the maximum battery capacity is divided by a parameter intended towards adding a buffer distance to hedge against the possible variations of the battery utilization. A branch-and-price approach is used to solve related vehicle scheduling models.

Only a few papers consider disruption management and robustness in integrated vehicle and crew scheduling including Lai and Leung (2018), Amberg et al. (2019) and Maenhout and Vanhoucke (2018). Integrated vehicle and crew scheduling in public transit may be enhanced in the context of robust resource allocation (Amberg et al. 2019). As already stated otherwise, integrated problem solving may result in more vulnerable and more fragile plans. That is, degrees of freedom are utilized to squeeze things in, e.g., for cost optimization or for whatever objective function is considered. Dependencies between scheduled vehicles and drivers may imply a major impact as small disturbances may easily propagate throughout an entire network. In Amberg et al. (2019) the authors investigate mutual dependencies between the different planning problems once handled in an integrated fashion and determine the propagation of possible delays. The goal of the paper is to show the impact of an integrated vehicle and crew scheduling approach by comparing sequential, partially integrated, and integrated vehicle- and crew-scheduling solutions. Numerical experiments regarding robustness and cost-efficiency are provided implying that incorporating possible delay propagation into the scheduling problem is useful and can be achieved in a cost-efficient way. Delay propagation in Amberg et al. (2019), Amberg (2017) follows fixed buffer times or a calculated measure that represents the possible propagation of results. Given a duty with a set of trips to be performed, a measure is defined incorporating expected primary delays and subsequent secondary delays. Using a simple means of robustness, Ge et al. (2022) incorporate delay propagation ideas from Amberg et al. (2019) into the model of Mesquita et al. (2013); it is shown that the original model as well as the extended robust one can now be solved with standard solvers for problem sizes that were bound to heuristics a decade ago.

As a first step towards more robustness in crew rostering, Xie et al. (2012) consider a simplified version of the classical crew rostering problem (called rota scheduling) but incorporate possible reserve shifts to cover the eventual absences of crew members (e.g. due to sickness). Doing so classifies this approach as belonging to the realm of anticipation. They formulate a two-stage stochastic model which assigns different shift types to the working days of the crew members, while coping with on-hand reserves and optional reserve shifts, too. The classical decision options are considered on stage one, and optional reserves are considered on stage two. As a solution approach the authors solve a deterministic equivalent with a standard MIP-solver.

In general, as mentioned above, the incorporation of reserve shifts or reserve duties may be considered as *anticipation*. These reserve duties would be available in case of disturbances if needed. As an example, Ingels and Maenhout (2015) investigate the implied robustness of anticipation measures under different scenarios.

A table with some recent references is given in the appendix (see Table 5).

### Delay management and delay propagation

Disturbances may cause delays and delays may result in passengers not arriving in time at their final destination or even earlier at intermediate stations that were supposed to be used as transfer stations. Despite all efforts in scheduling and schedule synchronization, a most important question is derived in this setting: “To wait or not to wait?” That is, delay management as it is used in literature concerns the issue of connecting trains having to wait or not to be able to pick up late transfer passengers. If the connecting train is connected to other trains or means of transport, this is again a cascading or propagation issue asking for a proper handling. That is, delay management is a part of disturbance management, not the other way around. A most comprehensive survey on delay management can be found in König (2020), Schöbel (2006) and Schmidt (2014). Related objectives may be different and mostly conflicting as we might consider minimizing passenger inconvenience or minimizing recovery time or minimizing cost implications. Beyond a recent literature review, König (2020) also attempts to provide a new classification of the field.

Closely related to delay management are also *mitigation strategies*. Characterizing delay management on a timeline classifies it as an operational problem rather than a tactical or strategic one. Usually the idea is to minimize passenger inconvenience. This is somewhat badly defined but broad enough to possibly result in interesting optimization problems which tend to be different from the quest of returning as quickly as possible to an originally given schedule and avoid further delay propagation in the network. Loosely spoken, this latter topic refers to the train perspective and refers to issues of (real-time) rescheduling where train delays are minimized. On the other hand, delay management aims at minimizing weighted passenger delay. Mostly, we see a macroscopic perspective which can be modeled by means of an event-activity network. Resulting MIP formulations can usually be solved with commercial solvers. However, the modeling may be enhanced in the interest of delayed passengers, taking into account rerouting; this, however, mostly increases the complexity of the resulting problem but improves the situation for some passengers.

Measures characterizing a disturbance and its influence (like the robustness indicators of Friedrich et al. (2017) mentioned earlier) can also be used as decision support measures in delay management. That is, if data is available regarding the number of possibly delayed passengers, this data can be used to make decisions regarding the above question with respect to waiting (or not). If all passenger data (like those related to OD pairs) would be known, the problems might be easy, but missing data and uncertainty makes this a challenging area. (Note that data may be missing on purpose due to data security measures.)

Taking a passenger’s view, trust in delay management seems not always very high. Passengers may assign a disutility to travel time uncertainty. In possibly non-scientific terms, this disutility is an anxiety cost for the necessity to proactively think about possible contingency plans in case of disruptions (Parbo et al. 2016).

*Delay propagation* is mostly used to address secondary delays arising based on decisions in delay management. Often event-activity networks are elaborated to investigate secondary delays. Moreover, according to Dollevoet et al. (2018), who provide a comprehensive treatment of delay propagation issues, delay management is mostly based on deterministic models, while the delays themselves are usually stochastic in nature. The view on delay propagation mostly seems to stem from the perspective of trains or buses that are possibly running late. A different view, like coming from the perspective of the passenger, the perspective of personnel or from the perspective of general infrastructure, trips or alike seem quite scarce in literature. Behavioral issues can be incorporated in mathematical modeling in different ways. In Schöbel et al. (2019) the situation of transport means (e.g. trains) being unable to depart due to (late-coming) passengers from other possibly delayed vehicles is investigated. These passengers are assumed to “trickle” in one after another, such that the doors of the departing vehicle cannot close. A mathematical programming approach is presented.

An important part of delay propagation seems the inclusion of buffer times or time supplements (Zieger et al. 2018; Jovanović et al. 2017; Lee et al. 2017; Amberg et al. 2019; Beśinović et al. 2016; Vansteenwegen et al. 2016; Dewilde et al. 2013; Ghaemi et al. 2018a; Ge et al. 2022). Rather than answering the question whether to wait, this also relates to incorporating fixed as well as variable buffer times upfront as part of the various planning stages. Beyond fixed buffer times the use of simulation-based optimization as well as robust optimization may be options that could be investigated further.

### Short turning

A common strategy in public transport (and especially in case of disturbances) is to have some means of transport not necessarily serving the full line, but to turn before reaching a terminus, possibly running back in the opposite direction (or even serving a different line or route). This is usually called *short turning* or, more seldom, turn back or even cut route. Short turning requires the availability of an appropriate facility (like a loop or related streets/tracks) or, e.g., appropriate cross-tracks and doorways on both sides of a tram or train to allow for related lay over and provision of service along the line.

Besides using short turning as a regular way to build schedules (like short turning to serve more frequently used parts of a line while providing thinned-out service in areas with low demand) it can be useful in case of disturbances. Assuming that some part of a line is closed due to some disturbance, the blocked vehicles may short turn and serve the non-affected part of the line, while the blocked part can be accommodated by means of bus bridging (see Sect. 5.6).

In Weerawat and Chumkad (2018), a short turn operation is proposed to cope with demand imbalances. In this case, different headways to prevent possible delays need to be considered. Different problem settings are possible, distinguishing the type of railway line, the infrastructure availability, etc. Inserting special short-turning services with the aim of achieving higher frequencies on certain segments is investigated in Canca et al. (2016). A more classical description can be found in Tirachini et al. (2011).

Different types of short turning may be distinguished depending, among others, on the time of its use (like immediately after a disturbance has occurred, during an ongoing disturbance, and shortly before a disturbance diminishes). In Chu and Oetting (2013) parameters are proposed which allow modeling the capacity consumption of turning stations during the transition phase of a DRP. A MIP model being able to solve real cases can be found in Ghaemi et al. (2018a). A connection of short turning with the impact of predictions on the length of disturbances is provided in Ghaemi et al. (2018b). Short turning may also be used in connection with bus bunching as investigated in Tian (2021) and Tian et al. (2022). In Yuan et al. (2022) an integrated optimization model for train timetabling, rolling stock assignment, and a short-turning strategy on a bidirectional metro line is investigated and verified for two case studies, a simplified metro line and a metro line in Beijing (China).

### Bus bridging

Bus bridging is applied in situations where rail-based disruptions are occurring (unexpectedly or even in a pre-planned way) and buses are used as replacement service to somehow re-establish transport network connectivity. Metro or rail disruption management is called bus bridging by some (e.g., Kepaptsoglou and Karlaftis 2009) and just uses ideas from bus bridging by others (e.g., Zhang and Lo 2018; Malucelli and Tresoldi 2019). Bridging refers to the idea that portions of the network must be reconnected; in case of subway or rail-based systems this often happens by means of extra bus services. Usually, the idea is to establish short-term bus routes to restore connectivity between stations affected by a disruption. Following Kepaptsoglou and Karlaftis (2009), the problem is to optimally design a bus bridging route network. This can be operationalized, e.g., by means of passenger welfare subject to given demand patterns, resource availability as well as route and service constraints. Maximizing passenger welfare may include appropriate or available capacity, low travel times and an immediate initiation of service through the assignment of enough buses to the substitute service. Different problem settings can aim to minimize costs while fulfilling a certain demand and other restrictions.

References regarding bus bridging also include the following: Jin et al. (2016), Liang et al. (2019) and Pender et al. (2015). When evaluating delays due to a disturbance, Aboudina et al. (2021) include direct delays due to the disturbance as well as indirect delays of bus riders on the routes from which shuttle buses are dispatched. A related case study is provided for Toronto (Canada). With a different flavor than most other studies, Zhang and Lo (2020) focus on an academically mostly neglected topic: setting up a contract between a mass transit provider (e.g. a company running a metro system) and a bus company providing the bridging service (whenever needed). A recent survey on the topic of bus bridging can be found in Zhang et al. (2021).

As mentioned before, a survey based on semi-structured interviews of 71 transit companies/agencies by Pender et al. (2013) provides insights regarding policies undertaken in practice. Along with common-sense considerations, most agencies used spare buses as bus-bridging vehicles. Only 45% of them actively retracted buses from existing scheduled bus services. An interesting question is also related to the location of facilities for spare buses potentially serving bridging activities. While these are usually the same as the given depots, this need not be the case in general (Pender et al. 2014a). It should be noted that beyond buses the use of taxis for bridging purposes also serves as an option (Fang and Jiang 2019; Fang et al. 2020). Asking for a collaboration effort with a taxi company to account for short-term tram disruptions is described in Zeng et al. (2012).

The above-mentioned studies usually assume a fixed assignment of buses to specific bridging routes, eventually with given frequency or headways. If more than one bridging route is concerned, one may certainly apply any type of scheduling approach, including the flexible serving of different bridging routes; see, e.g., Gu et al. (2018). In Christoforou et al. (2016) bus bridging is investigated in connection with other measures including an extension of services on alternative routes. A case study regarding an incident in Paris, France, in 2015 was used to exemplify this. The use of bus bridging in case of maintenance work is also possible; see, e.g., van der Hurk et al. (2016), who call it shuttle service. Using smart card data to enable efficient bus bridging is proposed in Luo et al. (2019).

Sometimes bus bridging is also used in the context of pure evacuation needs. For instance, Goerigk and Grün (2014) consider a specific bus evacuation problem, which is a vehicle scheduling problem that aims at minimizing the network clearance time, i.e., the time needed until the last person is brought to safety. While this may be a viable option in the sense of realizing a contingency plan (Janarthanan and Schneider 1984), this may also be misunderstood in relation to what the problem really is (e.g., Hu et al. (2016) mix pure bridging efforts with evacuation to result in a false impression of what they really do). Of course, one may see this concept in the spirit of the idea to move stranded passengers from affected (metro etc.) stations (which is different than “bridging” to keep up movements between effected stations during disruption). As Kepaptsoglou and Karlaftis (2009) seems to be most influential, their idea of applying genetic algorithms to solve related problems (with different objectives and constraints) is re-used (e.g. Hu et al. 2016).

Table 6 in the appendix summarizes some works on bus bridging. Note that case studies in the mentioned papers are usually hypothetical.

### Bus bunching

Bunching usually refers to two or more transport means of the same line following close to each other unintentionally. While bus bridging, literally, describes a solution approach for coping with certain types of disturbances, bus bunching in most situations, firstly, refers to a problem. That is, bus bunching is mostly described as a phenomenon. For some reason a vehicle is delayed implying an increased headway which may lead to more passengers to arrive at the stop or station who take more time to enter, which results in an even larger delay. The next vehicle might travel faster as less passengers get aboard. Close to entering a vicious cycle, eventually these two vehicles will bunch into each other, i.e., follow each other in very close distance. Solution approaches trying to avoid bunching then focus on minimizing the bunching advent and keeping the headways within pre-defined boundaries or minimizing the deviation from a given headway (or schedule). This can broadly be done in two directions. Firstly, for schedule-based approaches one gives schedule adherence highest priority and related measures have to be taken. Secondly, headway-based approaches try to take measures to keep the headways within certain boundaries, even if the schedule is not adhered to. The first is preferred in case that headways are larger, while the latter applies to cases with shorter headways (e.g., discriminating smaller or larger values than ten minutes for buses). In both cases, holding can be an acceptable approach; see, e.g., Berrebi et al. (2018). This may be the case if a tendency of running before schedule should be avoided or, if a certain headway needs to be adhered to, even if a previous vehicle is delayed. If speed variation is possible, this can be a reasonable approach to allow adhering to intended times. Another idea especially for expediting late vehicles could be skipping some stops with the disadvantage of possibly disappointing left-behind passengers. On the other hand, one may even skip certain services so that passengers might have to wait for a later one (Gao et al. 2016). Sometimes this is called leapfrogging (Nesheli and Ceder 2014).

Different methodologies can be found when tackling bunching. Examples for using a MPC approach to avoid bunching are Andres and Nair (2017) and Varga et al. (2019) (see Sect. 4.4 for a brief introduction to this type of approach). Andres and Nair (2017) combine a data-driven headway prediction with dynamic holding strategies. Data from an Irish bus route is used (i.e. Dublin). A real-time control strategy in the context of bus bunching can be found in Hernández et al. (2015).

For further studies on bus bunching see, e.g., Daganzo (2009), Bartholdi and Eisenstein (2012), Chandrasekar et al. (2002), He (2015), Iliopoulou et al. (2020), Saw et al. (2019) and de Souza and Sebastiani (2021). The most commonly used idea is to measure headway adherence at certain points and to possibly take some actions (e.g. waiting). Academic literature may be classified regarding those works that analyse bunching (see, e.g., Sun et al. 2021; Gong et al. 2020 for headway-based as well as smart card data-based prediction approaches) versus those that support planning and operations to avoid bunching. An important distinction of available information relates to local versus global information; see, e.g., Wang and Sun (2020). In the first case important information might be missing while the latter may be bound to information proliferation. A meaningful distinction in the analysis of the bunching phenomenon relates to the question whether overtaking is allowed; see, e.g., Fonzone et al. (2015) and Wu et al. (2017). This becomes even more important if a certain corridor is served by more than one line. In Schmöcker et al. (2016) this is investigated with the outcome that common lines have positive effects when overtaking is possible.

Different types of simulation models to explain and/or cope with bunching are provided in Gershenson and Pineda (2009). A nonlinear optimal control problem formulation to support the reduction of possible bus bunching is formulated by Li et al. (2019) and solved by means of a simplified convex optimization problem. The overall settings consider a pre-specified uncertainty set with influencing factors including disturbances due to delays as well as passenger demand uncertainties. In Petit et al. (2019) a bus substitution strategy is investigated where standby buses are dispatched to enhance system reliability. This may be meaningful especially in case of multiple lines.

While up to now we classified bus bunching as a problem, occasionally this can be viewed differently. Rather than being a problem but a planned situation, one can resort to something often called *platooning* (and not bunching), that is, the planned connection of several vehicles of one line (or even multiple lines). This can be found in seldom cases in some public transport systems worldwide and will become more important once autonomous vehicles are used on a wider scale (see, e.g., Sethuraman et al. 2019 and Nguyen et al. 2019b). The latter incorporates a simple means of delay management into their consideration.

Table 7 in the appendix summarizes some works on bunching.

### Maintenance and repair

Despite the fact that public transport infrastructure is vulnerable and bound to disturbances, it also needs to be regularly inspected, and maintenance and repair activities need to be scheduled to keep up the functionality of this infrastructure. Occasionally this is called *planned engineering* (Shires et al. 2019). While vehicles and many other things can be maintained and eventually repaired without causing visible disturbances to the customer, especially guided and tracked systems need to maintain their functionality and availability in a way that may be visible to the passenger. The interplay of timetables and maintenance is usually based on the idea that both can be fixed as well as variable leading to various problem settings. Especially in rail operations this may also account for a comprehensive interplay of passenger and freight train movements.

Preventive maintenance can often be scheduled so as not to interfere with regular operation, i.e., in many situations some tasks may be processed over night when related parts of the system are not in use. However, this is not always possible implying that occasionally large maintenance or renewal measures need to be done during daytime. In those cases, one may envisage the blocking of parts of the infrastructure for certain periods of time, e.g. hours, days or even more. Problem settings often combine strategic up to operational planning, as long-term infrastructure decisions may reduce the operational needs for maintenance.

Most of the available literature in this area relates to rail operations. In Arenas et al. (2018), the authors provide a MIP model that rearranges a timetable to cope with maintenance-activity-based capacity consumption. Besides the maintenance trains themselves, this also concerns temporary speed limitations for the related network part(s). A short-term application for a part of the French railway network, more specifically a section of the Paris—Le Havre line incorporating mixed traffic including intercity, regional, high speed and freight trains, is provided.

In Kiefer et al. (2018), the authors investigate renewal and maintenance activities that have to be performed in the long run. Given a certain planning horizon, all required activities have to be scheduled. Performing the same or similar activities on adjacent segments may imply cost savings, so that related planning may be beneficial. Moreover, workforce needs to be scheduled with different cost measures during the day or during the night. An optimization problem is formulated minimizing total costs, including those for maintenance work, replacement services, and additional vehicles. Linking bus bridging and maintenance and repair is a topic considered in van der Hurk et al. (2016).

A service-oriented objective is usually concerned with the service level offered to the passengers as can be encountered, e.g., in Vansteenwegen et al. (2016) and Louwerse and Huisman (2014). An important distinction is the cyclicity of the approaches, mostly related to the question whether the maintenance horizon is much longer than a typical timetable period. Cyclicity means a new schedule incorporating the maintenance activity as if it would be a regular service, while non-cyclic approaches aim to adjust the train scheduling before, during and after possible track closures.

Having provided pointers to works based on different objectives naturally leads to the quest to consider multiple objectives. For instance, D’Ariano et al. (2019) formulate a bi-objective optimization problem with the objectives of minimizing the deviation from a scheduled plan and maximizing the number of aggregated maintenance works under stochastic disturbances. This leads to research on the interplay between scheduling train operations as well as planning maintenance works on the same infrastructure. The interesting focus of this work relates to a way of measuring the quality of the obtained integrated solutions regarding their robustness with respect to stochastic perturbations of the train travel times as well as the maintenance works. Pareto optimality is investigated. Some references focusing on sustainability issues in connection with maintenance in public transport are collected in Alawaysheh and Alsyouf (2018) and Alawaysheh et al. (2020).

A comprehensive treatment of the topic, mostly focusing on freight rail applications, can be found in Lidén et al. (2018) and Lidén (2020). They focus on the coordination of railway network maintenance and train traffic, especially under the assumption of investigating a cyclic integrated train service and railway maintenance planning problem with resource considerations. They formulate the problem by means of a MIP model where the settings are easily transferable from freight rail to passenger rail. An interesting case study for single-track planning in Sweden is discussed.

A brief overview of papers in this field can be found in Table 8 in the appendix.

While we have, so far, looked at disturbances impacting public transport, one may also ask the question the other way around. That is, could it happen that traffic congestion is caused by public transport and how should that be assessed? Of the few works in this respect, we highlight a recent survey by Nguyen-Phuoc et al. (2020). Actually, maintenance and repair as well as infrastructure development regarding public transport with related work in progress might be options where this could happen.

### Miscellaneous

Focusing on public transport planning and operations often assumes explicitly or implicitly given assumptions (as encountered in the previous subsections). Therefore, many of the issues considered in the classification provided in Table 1 above may resort to problem settings beyond the scope of this paper (e.g. driver fatigue or crime). Though, some examples may be considered as follows.

One part of information management is to provide the public transport user with static as well as dynamic information (Daduna and Voß 1996). Current information technology allows related planning, e.g., by checking data in real time. That is, technology is available, but often even the data is available; cf. GTFS data, see footnote 11 in Sect. 6 below. This enables efficient itinerary planning for customers (Zhang and Tang 2018; Redmond et al. 2020). A robust optimization approach to address this problem is provided in Zhang and Tang (2018). In Hua and Ong (2018), the problem of information provision and contagion in disruption scenarios is investigated. A modelling framework is proposed using an information-based dynamic user equilibrium method; evidence is provided for the case of Singapore.

Another example concerns the impact of disturbances on ergonomics, service provider loyalty or business reputation; see, e.g., Golightly and Dadashi (2017) for the latter. Accounting for the interplay of variation in infrastructure availability and total travel costs is an issue investigated in Tahmasseby and van Nes (2007).

In general, a stronger focus on certain trade-offs seems worth more elaboration. This may refer to spare capacities as well as redundancy of critical infrastructure (e.g., closely related to redundancy allocation; Caserta and Voß 2015) but also to (redundant) standby personnel, etc. As a generic issue one may consider a better integration of the supply side (e.g., infrastructure on a strategic level but also operational planning) and the demand side and their influencing components, e.g., affected by the pricing or availability. An example is provided in Zhang et al. (2019), who investigate a revenue-maximization model integrating dynamic ticket-pricing, elasticity in passenger demand, and flexible dispatching (with a case study for the Guangzhou-Shenzhen (China) railway).

## Case studies

Robustness in public transport, as we have seen it, comes along with a wealth of different ideas and concepts. Many of the cited papers are providing numerical results, often on synthetic data, but occasionally on real-world cases. While sometimes the distinction is not necessary, we like to point out that the lessons learned from any of these studies depend on their settings. In that sense, we may deduce important implications from simulated studies as well as from real ones. In this section, we select a subset of the studies that may be important for future research in one way or another.^11^

Firstly, many of the different concepts pointed out in this paper have been considered in various real-world settings, some of which are exemplified in Table 9 in the appendix. A comprehensive table, mostly with earlier studies, can also be found in Rahimi et al. (2019). They display some 24 studies, most of which are considering pre-planned disruptions.

Simulation studies are available for various settings, including recurrent ones (like everyday-traffic-flow with congestion due to demand peaks) as well as non-recurrent ones (like terrorist attacks). Let us exemplify. Simulating non-recurrent events like terrorist attacks have been investigated by Angeloudis and Fisk (2006). They simulated a high-connectivity low-degree network showing that robustness with respect to a terrorist attack can be at least as good as scale-free systems. An obvious implication is that a system with a large portion of shared tracks is less robust than dedicated line systems which have a similar size. Considering the cases of Madrid (Spain) in March 2004 and London (UK) in July 2005 (non-recurrent terrorist attacks on metro systems), it seems that measures like bus bridging can have a major impact to restore the system’s connectivity to about 95 % very quickly.

A good information management application should possibly be able to support passenger flow guidance to alleviate recurrent congestion in urban rail transit networks. One may even think of formulating an optimization problem regarding where, when and what type of guidance information should be released to passengers to enable a smoother movement of people in crowded areas. If this favorably impacts passenger travel behavior, the attractiveness of those systems may be greatly enhanced. Providing related information at selected time intervals can benefit and save quite some amount of passenger travel time during peak-hours. In Yin et al. (2019) this is exemplified for the case of Beijing (China). Classical navigation systems are usually applied for individualized traffic, like in automotive navigation systems or for individuals using a mobile phone as a substitute for a map, both being based on modern satellite navigation systems.

A reference model for comprehensively implementing processes within public transport companies can be found in Scholz (2012). Even if their *disturbance management* is part of a separate model not included in the core application, this gives good insights. One of the popular systems for public transport companies around the world is the Hastus system (see, e.g., https://www.giro.ca/en-ca/our-solutions/hastus-software/hastus-for-schedulers/) which also includes robustness like measures for detour and alike.

A case study of using autonomous vehicles in public transport is provided in Riener et al. (2020).

## Conclusions and future research

In this paper we have surveyed the different strands of literature on managing disturbances, disruptions, delays and alike up to issues of robustness and reliability in public transport. Preventive and reactive actions can go in line with related recovery approaches as well as anticipation. This could be devoted to individual as well as integrated problem settings. Methodology-wise this realm uses heuristics, metaheuristics hybridized with mixed-integer programming, but also discrete event simulation and stochastic optimization.

Often, these strands of literature are not well connected and future research should be devoted to crossing the lines. Moreover, investigating matheuristics (Maniezzo et al. 2009) seems a major step forward regarding advances in methods for problem solving as envisaged in other areas (e.g. Doi et al. 2018). The same might hold for multi-criteria optimization, where borrowing from the airline industry may be beneficial (see, e.g., Ehrgott and Ryan 2002 for crew scheduling and robustness). Transferring approaches from the airline industry and supply chain risk management might be one successful way of moving this area forward. That is, there are quite a few studies investigating robustness in other areas like the airline industry. For instance, Ionescu and Kliewer (2011) distinguish stability and flexibility where the first is specified as the ability of a schedule to keep its feasibility as well as its cost-efficiency under different scenarios. Moving this into the direction of public transport is done in Amberg et al. (2017). This is especially interesting, as future research might investigate complexity issues related to different underlying networks (not only between different industries but also within the public transport domain itself). That is, applying known results from graph theory, the study of communication networks and related connectivity measures (e.g. using spectra of graphs) should be enhanced, too.

In a different direction, one of the open questions for future research refers to possible key performance indicators related to the various issues treated in this paper. This may concern the objectives to be considered in mathematical programming approaches. Rather than focusing on indicators regarding transit companies, indicators regarding passengers and passenger loyalty should be moved more into the foreground. Moreover, if at all, according to which indicators and in which way should we “penalize” changes to given plans once we need to perform replanning steps? Other issues need observance, too. Concerned are, for instance, passenger information systems to inform customers and workforce about possible implications of disturbances and also to give a prioritization related to different indicators and resources in case of conflicts (e.g. coping with financial limitations regarding scarce resources). Empirical studies and customer surveys might lay the foundation for related decision support. Among the very few examples attempting to provide meaningful answers in this respect is Grotenhuis et al. (2007). They state, e.g., that “Customers who use public transport frequently for study purposes express a greater need for an overview of routes by a multi-modal journey planner, but less need for route advice to avoid delays or disturbances.” Examples of related questionnaire-based or stated preference-based studies can be found in Currie and Muir (2017), Auld et al. (2020) and Rahimi et al. (2019). In Abenoza et al. (2018) we, e.g., find the idiom vulnerability, though, without any explanation. In that sense we might also doubt the whole study as the sample profile might have been generated without proper understanding. Though, overall, this area gives rise to the question whether it is sufficient to have empirical studies to understand the needs of passengers that are traveling while disturbances and alike occur. Most papers in this area neglect this and also de Oña et al. (2012) and van Lierop et al. (2018), as most prominent examples of a meta-analysis or similar on this do not provide enough hints. Maybe, if the right – rather than almost always the same – questions would be asked, this would change. In different settings, though, the idea of questionnaire-based research becomes valid and useful. For instance, Gómez-Ortiz et al. (2018) undertake a study from a completely different angle and different population parameters than many other studies. They explore bus rapid transit (BRT) drivers in the city of Bogota (Colombia) based on psychosocial risk factors at work leading to increased stress and health problems. Based on this study, measures may be taken to reduce the accident rates, etc. of the Bogota BRT system.

On a different scale, we see studies, such as Brauner (2017), which provides a decision framework considering subjective effects of security measures based on empirical evidence (like customers’ acceptance of drawbacks/interferences to cope with robustness). It is the believe of Brauner (2017) that both, socio-economic and socio-technical aspects in the context of a public transport system, can be put into perspective to weigh the importance of various effects (objective and subjective) and costs of security measures. Ultimately, the aim of improved customer satisfaction might be achieved. While the question whether we really need empirical studies in this context might be somewhat misleading, it leads us to a wealth of important future issues. As we usually encounter limited resources being available to cope with disturbances, empirical studies might help us to understand how and where these limited resources should be applied and how to rank in case of conflicts. At present, this, however, is mostly not considered in academic literature in this field and needs respective effort.

Content-wise, future research could incorporate the notion of load-dependent lead times (see Pahl et al. 2007 for a survey of load-dependent lead times in production) into public transport. As an example, consider Fig. 3 in Sect. 2.2 above. In the sense of achieving solutions, common delays based on disturbances might be included, like load-dependent travel times in peak hours in case that historical data reveals a certain delay in peak hours with a certain probability. These probabilities might be based on expected load-dependent travel times and data-driven forecasting measures. On the other hand, if this is related to different traffic conditions or time-varying speed limits, it should become part of the data and not be related to disturbance. This topic needs to be explored further. While this might be in conflict with the idea to utilize capacity to the limit (see, e.g., Pellegrini et al. 2017), it possibly makes capacity utilization more robust.

Another recent issue for future research refers to curfew and related topics in case of a pandemic and political measures to avoid it. For instance, the financial situation of public transport companies might be most seriously influenced by such measures based on changing demand. On a different side, e.g., fare evasion might be a topic as the measures might imply safety distances between people that do not allow for proper checking and control. Possible solutions to be evaluated are tap-in tap-out machines with cameras or related robots. Moreover, following the recently observed pandemic situation requires quite a few drastic changes in public transport (Voß et al. 2020). For instance, if social distancing is followed in an appropriate way, then systems that are already beyond their limits might need even more capacity and infrastructure. Moreover, stations require different forms of queuing. Complying with social distancing and hygienic issues already applies during access as well as during transfer, implying a rethinking of station design as well as station maintenance.

Finally, the upcoming advent of electric vehicles as well as autonomous vehicles especially in connection with MaaS (Mobility-as-a-Service; see Wong et al. (2020) for a recent survey) provide a wealth of issues worth investigating. For instance, the usability of batteries and related time and length restrictions for those vehicles need to be observed in a different way than for usual solutions. In case of MaaS, despite data safety regulations, providing sufficient information could be used on an individualized rather than collective basis to overcome disturbances (especially for handicapped people this might be very useful). Literally, one may think of automatically coping with disturbances.

## Appendix

This appendix includes quite a few tables summarizing some of the discussed topics and cited works (Tables 3, 4, 5, 6, 7, 8, 9).

**Table 3:** Selected/representative literature on network design under disturbances

References	Focus	Comments
Cats and Jenelius (2015), Cats (2016)	Capacity increase	Case of Stockholm (Sweden)
Ibarra-Rojas et al. (2015)	Literature review up to 2015	Emphasis on real-time control strategies suitable to bus transport systems
Iliopoulou and Kepaptsoglou (2021)	Robust optimization and metaheuristic approaches	Emphasis on electric bus networks
Nian et al. (2019)	Metro, Shanghai (China)	Alignment corridor of a new metro line considering network vulnerability
Peled et al. (2019)	Transit network design and frequency setting problem	Predictive optimization
Yan et al. (2013), Yan et al. (2012)	Robust optimization	Bus transit network design with stochastic travel time
Yao et al. (2014)	Stochastic travel times	Satisfying passenger demand and reliable transit service

**Table 4:** Selected/representative literature on timetabling under disturbances

References	Focus	Comments
Beśinović et al. (2016)	Microscopic and macroscopic network model	Hierarchical framework for timetable design
Beśinović et al. (2019)	Issues of capacity extensions and stable timetables	Heuristic model and MIP; case study for Netherlands railways
Bettinelli et al. (2017)	Train rescheduling	Iterated greedy heuristic
Cacchiani et al. (2012)	Aperiodic train timetabling problem	Lagrangian heuristic, case from the Italian Railways
Cacchiani and Toth (2018)	Comprehensive survey on methods for robust timetabling	
Fischetti and Monaci (2009)	Introduction of light robustness	
Khoshniyat and Peterson (2017)	Technical minimum headway plus some buffer time	MIP model; evaluated for Swedish rail
Louwerse and Huisman (2014)	Service-oriented objective, maximize the service level offered to the passengers	No explicit rolling stock rescheduling; MIP model; application to instances from Netherlands Railways
Lusby et al. (2018)	Comprehensive survey up to 2018	Emphasis regarding rail operations
Qi et al. (2018)	Robust integrated train timetabling and stop planning problem	Case study for Wuhan-Guangzhou (China)
Schöbel (2014)	Timetabling with light robustness	
Shakibayifar et al. (2018)	Train rescheduling	Simulation-optimization approach
Veelenturf et al. (2016a)	Minimize the number of cancelled and delayed train services	ILP; adhering to infrastructure and rolling stock capacity constraints
Vromans et al. (2006)	Reducing running time differences per track section	Attempt to design homogeneous timetables, heuristics, simulation
Yuan et al. (2022)	Integrated optimization model for train timetabling, rolling stock assignment, and a short-turning strategy	Case study for a metro line in Beijing (China)

**Table 5:** Selected/representative literature on vehicle and crew scheduling under disturbances

References	Focus	Comments
Amberg et al. (2019), Amberg (2017)	Integrated vehicle and crew scheduling	Delay propagation using fixed buffer times and a calculated measure
Cavone et al. (2019)	Bi-level rescheduling	MIP model, case study for rescheduling Dutch railway traffic
Ge et al. (2022)	Integrated vehicle and crew scheduling	Delay propagation as well as replication studies
Lai and Leung (2018)	Integrated vehicle and crew scheduling	Stochastic and time-dependent travel times
Maenhout and Vanhoucke (2018)	Integrated personnel shift and task rescheduling problem	Heuristic rescheduling procedure
Potthoff et al. (2010)	Set covering formulation for the problem of crew rescheduling	Nested neighborhoods, MIP model
Veelenturf et al. (2012)	Crew rescheduling with retiming (the latter means to allow for small modifications/delays of the times)	Column generation with Lagrangian heuristics
Verhaegh et al. (2017)	Results for instances based on the Dutch railways	Crew rescheduling for small disruptions with an insertion heuristic and an iterative-deepening depth-first search
Xie et al. (2012)	Crew rostering	Reserve shifts

**Table 6:** Selected/representative literature on bus bridging

References	Focus	Comments
Chen and An (2021)	Joint optimization of bus bridging routes and timetables under time-varying demand	Case study for Melbourne (Australia)
Gu et al. (2018)	Flexible serving of different bridging routes	Case study for Shanghai (China)
Jin et al. (2016)	Three-step optimization approach, demand-responsive candidate bus routes	Two cases
Kang et al. (2019)	Last train timetable optimization with bus bridging, MIP model and decomposition approach	Case for Vienna (Austria)
Kepaptsoglou and Karlaftis (2009)	Methodological framework for planning and designing a bus bridging network	Among the most cited works on bus bridging
Liang et al. (2019)	Multi-commodity flow model, robust optimization approach	Case studies for Beijing (China)
Liu et al. (2020)	Investigation of bus bridging policies and ride-hailing regarding equitable distribution of transit capacity	Analysis of disruptions in Toronto (Canada)
Luo and Xu (2021)	Consideration of uncertainty, stochastic programming model	Case study for Singapore
Pender et al. (2014a)	Strategically locating satellite bus reserves incorporating disruption likelihood	
Tang et al. (2021)	Linear programming model, focus on system resilience	Case studies for Singapore and Chongching (China)
Wang et al. (2019)	Multi-objective optimization under fleet size and vehicle capacity constraints	Case study for Shanghai (China)
Wang et al. (2014)	Compound Poisson processes, focus on different types of passengers	
Yang et al. (2017)	Metro system congestion and overcrowding mitigation through bus bridging	Case study for Shanghai (China)
Yin et al. (2018)	Three-layer discrete choice behavior model to analyze the dynamic passenger flow demand under station disruption	Case for Beijing (China)
Zhang and Lo (2020)	Contract design between a mass transit provider and a bus company providing the bridging service

**Table 7:** Selected/representative literature on bus bunching

References	Focus	Comments
Andres and Nair (2017)	Model-predictive control	Case study for Dublin (Ireland)
Arriagada et al. (2019)	Using bus GPS and AFC data to analyze factors associated with bunching	Cases for Santiago (Chile) and Gatineau (Canada)
Bartholdi and Eisenstein (2012)	Self-correcting method	Case study for Atlanta (GA, USA).
Berrebi et al. (2018)	Dynamic holding	Exemplification with TriMet (Portland, USA)
Daganzo (2009)	Dynamically determining holding times at a route’s control points	Among the most cited works on bus bunching
Degeler et al. (2021)	Define and determine bunching swings as repeating patterns of pairs of delayed and bunched vehicles	Case study for the Hague (Netherlands)
Drabicki et al. (2021)	Feedback loop based on real-time crowding information	Extended towards multiple-origins bus operation
Enayatollahi et al. (2019)	Cellular automata	Headway-based bunching index is proposed
Moreira-Matias et al. (2016)	Automatic control framework using a combination of various machine learning methods	Case Study for Porto (Portugal) over a period of 1 year
Nguyen et al. (2019a)	Determine factors related to the time of initial bunching incidents for a streetcar system	Case study using AVL data for Toronto (Canada)
Sajikumar and Bijulal (2021)	Schedule planning at certain entry points	Consideration of multiple-origins bus operation
Sethuraman et al. (2019)	Platooning	Impact analysis regarding traffic control and energy consumption
Sun and Schmöcker (2018)	Passenger behavior	Considering overtaking being allowed (or not)
Tian (2021)	Use of short-turning strategy to alleviate bus bunching	Case for Beijing (China)
Varga et al. (2019)	Model-predictive control	Includes a passenger waiting model
Verbich et al. (2016)	Combining AVL and APC data to investigate the connection of bunching with dwell and running times	Data from TriMet (Portland, USA)
Zhao et al. (2016)	Consideration of boarding limits for buses	Comparison with Bartholdi and Eisenstein (2012)

**Table 8:** Selected/representative literature on maintenance and repair

References	Focus	Comments
Arenas et al. (2018)	Rail, case study from French rail	MIP model to rearrange a timetable to cope with capacity consumption due to maintenance activities (maintenance trains, temporary speed limits etc.)
D’Ariano et al. (2019)	Rail	Bi-objective optimization, MIP model, short-term maintenance work
Kiefer et al. (2018)	Tram, case study in Vienna (Austria)	Strategic scheduling of preventive maintenance tasks; MIP model and large neighborhood search; possible connection to bus bridging
Lidén et al. (2018), Lidén (2020)	Rail, case study in Sweden	Coordination of railway network maintenance and train traffic, cyclic integrated train service and railway maintenance planning problem with resource considerations; MIP model; the settings are transferable from freight rail to passenger rail
Louwerse and Huisman (2014)	Rail, instances from Netherlands Railways	Integer programming; applicable in general, not only for maintenance
Su et al. (2017)	Rail	Scenario-based chance-constrained MPC
van der Hurk et al. (2016)	Case study in Boston, USA	Formulation of a shuttle design problem linking maintenance and repair with bus bridging
Vansteenwegen et al. (2016)	Rail	Timetable adaptation, good argument for sensitivity analysis

**Table 9:** Selected case studies

References	Transport system	Comment
Baghoussi et al. (2018)	Porto (Portugal), bus	Robust optimization model; slack times are used as decision variables to improve bus schedules
Bender et al. (2013)	Athens (Greece), metro	Online delay management, competitive analysis, stochastic optimization; comparison between online and optimal offline algorithm for delay management, worst case analysis and modifications
Caimi et al. (2012)	Bern (Switzerland), rail	Model-predictive control for a railway station area with rescheduling of trains
Candelieri et al. (2019)	Florence (Italy) and Attika (Greece)	Graph-theoretical measures on connectivity for attacks and cascading failures; includes betweenness centrality and related measures
Cats and Jenelius (2018)	Stockholm (Sweden), bus, metro, light rail	Dynamic agent-based modelling of network performance; gradual capacity reduction rather than complete failure, modified assignment model
Chen et al. (2014)	Guangzhou (China)	Comparison of different measures regarding network topological structure; graph-theoretical measures
Dridi et al. (2005)	Valenciennes (France), bus	Real-time control; decision-support system, Petri-net modeling
Ghaemi et al. (2018a)	The Netherlands, rail	MIP model for short turning
Gómez-Ortiz et al. (2018)	Bogota (Colombia), bus	BRT driver questionnaire to explore psychosocial risk factors at work
Huang et al. (2020)	Beijing (China), metro	Nonlinear mixed-integer programming models with different recovery strategies; may even include short turning
Kang et al. (2020)	Singapore, bus	Bus driver scheduling problem with uncertain bus travel times
Kiefer et al. (2016)	Vienna (Austria), tram and subway	maintenance and repair planning; MIP model, replacement line plan
Liebchen et al. (2010)	Germany, train network	Light robustness, delay resistant periodic timetables
Mahdavi et al. (2020)	New Delhi (India), bus	Index for dynamic resilience, also considering network complexity
Malucelli and Tresoldi (2019)	Milan (Italy), tram and bus	Discrete event simulation, tabu search, column generation; simulation-based optimization
Mudigonda et al. (2019)	New Jersey (USA), bus, rail and subway	Performance measures regarding the recovery after hurricane Sandy
Ng and Lo (2016)	HongKong (China), ferry	transit network design with limited data availability on passenger demand
Rietveld et al. (2001)	The Netherlands, multi-modal	Considers bike for the last mile; customer valuation of delay and alternative modes
Sparing and Goverde (2013)	West of the Netherlands, bus and train	Max-plus algebra; method from linear algebra to minimize transfer waiting times (cf. delay management)
Szymański et al. (2018)	Warsaw, Poland	Methodology to analyze the distribution of delays in public transport based on GPS data
Weerawat and Chumkad (2018)	Bangkok (Thailand)	Simulation of short turning
Xing et al. (2017)	Shanghai (China), subway	Vulnerability analysis; visualization, different measures like betweenness
Yin et al. (2019)	Beijing (China), subway	Flow guidance, information management; when should which information be provided in case of disturbance
Zhan et al. (2016)	Beijing and Shanghai (China)	Train rescheduling problem, MIP models, rolling horizon approach

## Footnotes

1 Note that we do not claim to be fully comprehensive as that would exceed the limits of this paper. Rather we aim at providing recent pointers that cover (almost) everything necessary when used in a proper backward search. That is, the text is interleaved with pointers to existing literatures without trying to follow some of the recent so-called structured literature review concepts or similar (see van Lierop et al. 2018; Brendel and Mandrella 2016; Risser et al. 2015; Chowdhury and Ceder 2016 as examples; those are mostly not available regarding our focus; an exception is Pender et al. (2014b)). That is, our approach may be best classified as *narrative* (Pahl and Voß 2022). Occasionally concepts are only briefly sketched, without providing the complete list of existing literature. Beyond our knowledge of the field and related literature we used a search on Scopus and Google Scholar on “public transport” and related phrases (like “public transport” and robustness) together with related backward and forward search. In most cases, the backward search was limited to the last five years. For a scientometric analysis of public transport research for 2009 up to 2013 see Heilig and Voß (2015).

2 Of course, we are aware of the ever rejuvenated discussion whether public transport should be provided for free—but even that is a price—to move people away from using private cars to reduce climate- and emission-related problems. This is long known especially since the discussion of Nobel price winner Vickrey about public transport in New York (USA) (Vickrey 1955, 1963). If pricing is concerned, behavioral aspects may come into place, too, e.g. regarding fare evasion (Barabino et al. 2020).

3 The notion of rich problems is more known in the vehicle-routing domain (Hartl et al. 2006), but can certainly be used in public transport, too. The connection is easily made if one considers the area of paratransit or demand-responsive transport as it includes the well-known class of dial-a-ride problems; see, e.g., Molenbruch et al. (2017) and Parragh et al. (2008) for surveys.

4 Having defined all these settings, we should note in passing that there are also other types of public transport that might not necessarily be covered in this paper, as they regard, e.g., to paratransit in a different meaning than before. *Paratransit* modes can be seen as systems between public transit and individual motorized traffic in terms of providing demand-responsive services even without a pre-defined route or pre-defined schedules, as it can be found especially in many third-world countries and even as part of the shadow economy in many places.

5 An interesting investigation regarding pandemic implications for Stockholm, Sweden, is reported on https://people.kth.se/~jenelius/corona_pt/ (see also Almlöf et al. 2021) and in general on https://www.transformative-mobility.org/news/the-covid-19-outbreak-and-implications-to-public-transport-some-observations, last access March 18, 2022.

6 Beyond what was already stated, let us exemplify as food for thought. For instance, in Cats et al. (2017) we find a definition where “systems are considered robust based on their capacity to absorb disruptions with a minimal impact on system performance.” Here we could ask how “minimal” is meant as a formal or a colloquial word, respectively.

7 With a special focus regarding this subsection, we may refer to, e.g., Brouwer and Haemers (2011) and Cvetkovic et al. (1980).

8 Note that in supply chain risk management (Sodhi and Tang 2012), one often distinguishes different types of uncertainty eventually leading to disturbances. Operational uncertainty may refer to inherently uncertain parameters like demand. This may or may not cause a disturbance. Disruptive uncertainty or disruption refers to events causing disturbances.

9 We assume that the reader has some basic understanding about the notions of solution methods in OR so that not all need to be explained in detail. It is literally biased food for thought.

10 See, e.g., https://www.ibm.com/support/knowledgecenter/SSSA5P_12.10.0/COS_KC_home.html and http://www.gurobi.com/products/gurobi-optimizer/gurobi-overview.

11 In passing, we note that often research claims problems regarding the availability of real data. Usually, this should not be a concern as there are many real-world data available (Ge et al. 2021). As an example, the attempts of many companies to visualize their efforts in being in time are made public; see, e.g., the Zurich (Switzerland) data available under https://data.stadt-zuerich.ch/dataset/vbz_fahrzeiten_ogd. Related to this is also the General Transit Feed Specification (GTFS); see, e.g., https://developers.google.com/transit/gtfs-realtime, an effort of providing users with real-time transit information. Moreover, many academics provide data they gained or constructed when working on public transport projects. As an example consider https://personal.eur.nl/huisman/instances.htm. A list of aggregated BRT data and performance measures is provided at https://brt.fareast.mobi/. For additional access options see (National Academies of Sciences, Engineering, and Medicine 2015; Ge et al. 2021). Case studies using and possibly integrating different data and systems include Godfrid et al. (2022) for Buenos Aires (Argentina), Aemmer et al. (2022) for Seattle (USA), Nimpanomprasert et al. (2022) for Hamburg (Germany), and Sarhani and Voß (2022) for Canberra (Australia). Possible data to be integrated include those from automatic vehicle location (AVL), automatic fare collection (AFC) and automatic passenger counting (APC) systems as well as weather data, etc.

## References

[CR1] Abbink E, Fischetti M, Kroon L, Timmer G, Vromans M (2005) Reinventing crew scheduling at Netherlands railways. Interfaces 35(5):393–401. 10.1287/inte.1050.0158

[CR2] Abenoza RF, Ettema DF, Susilo YO (2018) Do accessibility, vulnerability, opportunity, and travel characteristics have uniform impacts on the traveler’s experience? Transp Res Part A Policy Pract 114:38–51. 10.1016/j.tra.2018.03.017

[CR3] Aboudina A, Itani A, Diab E, Srikukenthiran S, Shalaby A (2021) Evaluation of bus bridging scenarios for railway service disruption management: a users’ delay modelling tool. Public Transp 13:457–481. 10.1007/s12469-020-00238-w

[CR4] Adelé S, Tréfond-Alexandre S, Dionisio C, Hoyau PA (2019) Exploring the behavior of suburban train users in the event of disruptions. Transp Res Part F Traff Psychol Behav 65:344–362. 10.1016/j.trf.2019.08.009

[CR5] Aemmer Z, Ranjbari A, MacKenzie D (2022) Measurement and classification of transit delays using GTFS-RT data. Public Transp. 10.1007/s12469-022-00291-7

[CR6] Alawaysheh I, Alsyouf I, Tahboub Z, Almahasneh H (2020) Selecting maintenance practices based on environmental criteria: a comparative analysis of theory and practice in the public transport sector in UAE/DUBAI. Int J Syst Assur Eng Manag 11:1133–1155. 10.1007/s13198-020-00964-1

[CR7] Alawaysheh I, Alsyouf I (2018) Environmental sustainability in maintenance management of public transport systems: Literature review. In: IEEE international conference on industrial engineering and engineering management (IEEM). pp 1125–1129. IEEE. 10.1109/IEEM.2018.8607535

[CR8] Almlöf E, Rubensson I, Cebecauer M, Jenelius E (2021) Who continued travelling by public transport during COVID-19? Socioeconomic factors explaining travel behaviour in Stockholm 2020 based on smart card data. Eur Transp Res Rev. 10.1186/s12544-021-00488-0 (article 31) 10.1186/s12544-021-00488-0 PMC8180438 38624666

[CR9] Amberg B (2017) Robuste Effizienz des Ressourceneinsatzes im öffentlichen Personennahverkehr. Ph.D. thesis, Fachbereich Wirtschaftswissenschaft, Freie Universität Berlin

[CR10] Amberg B, Ionescu L, Kliewer N (2017) Robust efficiency in public bus transport and airline resource scheduling. In: Dörner KF, Ljubic I, Pflug G, Tragler G (eds) Operations research proceedings 2015. p 259–264. Springer, Cham, 10.1007/978-3-319-42902-1_35

[CR11] Amberg B, Amberg B, Kliewer N (2019) Robust efficiency in urban public transportation: minimizing delay propagation in cost-efficient bus and driver schedules. Transp Sci 53:89–112. 10.1287/trsc.2017.0757

[CR12] An K, Lo HK (2014) Ferry service network design with stochastic demand under user equilibrium flows. Transp Res Part B Methodol 66:70–89. 10.1016/j.trb.2013.10.008

[CR13] Andres M, Nair R (2017) A predictive-control framework to address bus bunching. Transp Res Part B Methodol 104:123–148. 10.1016/j.trb.2017.06.013

[CR14] Angeloudis P, Fisk D (2006) Large subway systems as complex networks. Physica A Stat Mech Appl 367:553–558. 10.1016/j.physa.2005.11.007

[CR15] Arenas D, Pellegrini P, Hanafi S, Rodriguez J (2018) Timetable rearrangement to cope with railway maintenance activities. Comput Oper Res 95:123–138. 10.1016/j.cor.2018.02.018

[CR16] Arriagada J, Gschwender A, Munizaga MA, Trepanier M (2019) Modeling bus bunching using massive location and fare collection data. J Intell Transp Syst 23:332–344. 10.1080/15472450.2018.1494596

[CR17] Auld J, Ley H, Verbas O, Golshani N, Bechara J, Fontes A (2020) A stated-preference intercept survey of transit-rider response to service disruptions. Public Transp 12:557–585. 10.1007/s12469-020-00243-z

[CR18] Bababeik M, Khademi N, Chen A (2018) Increasing the resilience level of a vulnerable rail network: the strategy of location and allocation of emergency relief trains. Transp Res Part E Logist Transp Rev 119:110–128. 10.1016/j.tre.2018.09.009

[CR19] Baggag A, Abbar S, Zanouda T, Srivastava J (2018) Resilience analytics: coverage and robustness in multi-modal transportation networks. EPJ Data Sci 7, 10.1140/epjds/s13688-018-0139-7 (paper no 14)

[CR20] Baghoussi Y, Mendes-Moreira J, Emmerich MTM (2018) Updating a robust optimization model for improving bus schedules. In: 10th International conference on communication systems networks (COMSNETS). pp 619–624. 10.1109/COMSNETS.2018.8328284

[CR21] Bai D, Carpenter T, Mulvey J (1997) Making a case for robust optimization models. Manag Sci 43(7):895–907. 10.1287/mnsc.43.7.895

[CR22] Barabino B, Lai C, Olivo A (2020) Fare evasion in public transport systems: a review of the literature. Public Transp 12:27–88. 10.1007/s12469-019-00225-w

[CR23] Bartholdi JJ, Eisenstein DD (2012) A self-coördinating bus route to resist bus bunching. Transp Res Part B Methodol 46:481–491. 10.1016/j.trb.2011.11.001

[CR24] Bell MG, Kurauchi F, Perera S, Wong W (2017) Investigating transport network vulnerability by capacity weighted spectral analysis. Transp Res Part B Methodol 99:251–266. 10.1016/j.trb.2017.03.002

[CR25] Bender M, Büttner S, Krumke S (2013) Online delay management on a single train line: beyond competitive analysis. Public Transp 5:243–266. 10.1007/s12469-013-0070-z

[CR26] Berche B, von Ferber C, Holovatch T, Holovatch Y (2009) Resilience of public transport networks against attacks. Eur Phys J B 71(1):125–137. 10.1140/epjb/e2009-00291-3

[CR27] Berrebi SJ, Hans E, Chiabaut N, Laval JA, Leclercq L, Watkins KE (2018) Comparing bus holding methods with and without real-time predictions. Transp Res Part C Emerg Technol 87:197–211. 10.1016/j.trc.2017.07.012

[CR28] Beśinović N (2020) Resilience in railway transport systems: a literature review and research agenda. Transp Rev 40:457–478. 10.1080/01441647.2020.1728419

[CR29] Beśinović N, Goverde RM, Quaglietta E, Roberti R (2016) An integrated micro-macro approach to robust railway timetabling. Transp Res Part B Methodol 87:14–32. 10.1016/j.trb.2016.02.004

[CR30] Beśinović N, Quaglietta E, Goverde RM (2019) Resolving instability in railway timetabling problems. EURO J Transp Logist 8:833–861. 10.1007/s13676-019-00148-3

[CR31] Bettinelli A, Santini A, Vigo D (2017) A real-time conflict solution algorithm for the train rescheduling problem. Transp Res Part B Methodol 106:237–265. 10.1016/j.trb.2017.10.005

[CR32] Binder S, Maknoon Y, Bierlaire M (2017) The multi-objective railway timetable rescheduling problem. Transp Res Part C Emerg Technol 78:78–94. 10.1016/j.trc.2017.02.001

[CR33] Blenkers L (2015) Railway disruption management. Master thesis, Faculty of Mechanical, Maritime and Materials Engineering, Delft University of Technology. https://repository.tudelft.nl/islandora/object/uuid:37d16ba2-ced7-47c0-9155-2eb257cee0eb/datastream/OBJ/download

[CR34] Borndörfer R, Langenhan A, Löbel A, Schulz C, Weider S (2013) Duty scheduling templates. Public Transp 5:41–51. 10.1007/s12469-013-0064-x

[CR35] Braess D (1968) Über ein Paradoxon aus der Verkehrsplanung. Unternehmensforschung 12:258–268. 10.1007/BF01918335

[CR36] Brauner F (2017) Securing public transportation systems: an integrated decision analysis framework for the prevention of terrorist attacks as example. Springer, Wiesbaden. 10.1007/978-3-658-15306-9

[CR37] Brendel AB, Mandrella M (2016) Information systems in the context of sustainable mobility services: A literature review and directions for future research. In: Proceedings of the twenty-second Americas conference on information systems (AMCIS), San Diego

[CR38] Brouwer AE, Haemers W (2011) Spectra of graphs. Springer, Berlin. 10.1007/978-1-4614-1939-6

[CR39] Bruglieri M, Bruschi F, Colorni A, Luè A, Nocerino R, Rana V (2015) A real-time information system for public transport in case of delays and service disruptions. Transp Res Procedia 10:493–502. 10.1016/j.trpro.2015.09.003

[CR40] Bruyelle JL, O’Neill C, El-Koursi EM, Hamelin F, Sartori N, Khoudour L (2014) Improving the resilience of metro vehicle and passengers for an effective emergency response to terrorist attacks. Saf Sci 62:37–45. 10.1016/j.ssci.2013.07.022

[CR43] Cacchiani V, Caprara A, Fischetti M (2012) A Lagrangian heuristic for robustness, with an application to train timetabling. Transp Sci 46:124–133. 10.1287/trsc.1110.0378

[CR44] Cacchiani V, Huisman D, Kidd M, Kroon L, Toth P, Veelenturf L, Wagenaar J (2014) An overview of recovery models and algorithms for real-time railway rescheduling. Transp Res Part B Methodol 63:15–37. 10.1016/j.trb.2014.01.009

[CR41] Cacchiani V, Toth P (2012) Nominal and robust train timetabling problems. Eur J Oper Res 219:727–737. 10.1016/j.ejor.2011.11.003

[CR42] Cacchiani V, Toth P (2018) Robust train timetabling. In: Borndörfer R, Klug T, Lamorgese L, Mannino C, Reuther M, Schlechte T (eds) Handbook of Optimization in the Railway Industry. Springer, Cham, pp 93–115. 10.1007/978-3-319-72153-8_5

[CR45] Cadarso L, Marín A (2014) Recovery of disruptions in rapid transit networks with origin-destination demand. Procedia Soc Behav Sci 111:528–537. 10.1016/j.sbspro.2014.01.086

[CR46] Caimi G, Fuchsberger M, Laumanns M, Lüthi M (2012) A model predictive control approach for discrete-time rescheduling in complex central railway station areas. Comput Oper Res 39:2578–2593. 10.1016/j.cor.2012.01.003

[CR47] Canca D, Barrena E, Laporte G, Ortega F (2016) A short-turning policy for the management of demand disruptions in rapid transit systems. Ann Oper Res 246:145–166. 10.1007/s10479-014-1663-x

[CR48] Candelieri A, Galuzzi B, Giordani I, Archetti F (2019) Vulnerability of public transportation networks against directed attacks and cascading failures. Public Transp 11:27–49. 10.1007/s12469-018-00193-7

[CR49] Caschili S, Medda FR, Reggiani A (2015) Guest editorial: Resilience of networks. Transp Res Part A Policy Pract 81:1–3. 10.1016/j.tra.2015.07.010

[CR50] Caserta M, Voß S (2009) Metaheuristics: intelligent problem solving. In: Maniezzo V, Stützle T, Voß S (eds) Matheuristics: Hybridizing Metaheuristics and Mathematical Programming. Springer, Boston, pp 1–38. 10.1007/978-1-4419-1306-7_1

[CR51] Caserta M, Voß S (2015) An exact algorithm for the reliability redundancy allocation problem. Eur J Oper Res 244:110–116. 10.1016/j.ejor.2015.01.008

[CR52] Caserta M, Voß S (2020) A general corridor method-based approach for capacitated facility location. Int J Prod Res 58:3855–3880. 10.1080/00207543.2019.1636320

[CR53] Cats O (2016) The robustness value of public transport development plans. J Transp Geogr 51:236–246. 10.1016/j.jtrangeo.2016.01.011

[CR54] Cats O, Jenelius E (2015) Planning for the unexpected: the value of reserve capacity for public transport network robustness. Transp Res Part A Policy Pract 81:47–61. 10.1016/j.tra.2015.02.013

[CR55] Cats O, Jenelius E (2018) Beyond a complete failure: the impact of partial capacity degradation on public transport network vulnerability. Transportmetrica B Transp Dyn 6(2):77–96. 10.1080/21680566.2016.1267596

[CR56] Cats O, Koppenol GJ, Warnier M (2017) Robustness assessment of link capacity reduction for complex networks: application for public transport systems. Reliab Eng Syst Saf 167:544–553. 10.1016/j.ress.2017.07.009

[CR57] Cavone G, Blenkers L, van den Boom T, Dotoli M, Seatzu C, De Schutter B (2019) Railway disruption: a bi-level rescheduling algorithm. In: 6th International conference on control, decision and information technologies (CoDIT). pp 54–59. 10.1109/CoDIT.2019.8820380

[CR58] Ceder A (2015) Public transit planning and operation, 2nd edn. CRC, Boca Raton

[CR59] Çetinkaya E, Alenazi M, Peck A, Rohrer JP, Sterbenz JPG (2015) Multilevel resilience analysis of transportation and communication networks. Telecommun Syst 60:515–537. 10.1007/s11235-015-9991-y

[CR60] Chandrasekar P, Cheu RL, Chin HC (2002) Simulation evaluation of route-based control of bus operations. J Transp Eng 128(6):519–527. 10.1061/(ASCE)0733-947X(2002)128:6(519)

[CR61] Chen Y, An K (2021) Integrated optimization of bus bridging routes and timetables for rail disruptions. Eur J Oper Res 295:484–498. 10.1016/j.ejor.2021.03.014

[CR62] Chen S, Claramunt C, Ray C (2014) A spatio-temporal modelling approach for the study of the connectivity and accessibility of the Guangzhou metropolitan network. J Transp Geogr 36:12–23. 10.1016/j.jtrangeo.2014.02.006

[CR63] Chowdhury S, Ceder A (2016) Users’ willingness to ride an integrated public-transport service: a literature review. Transp Policy 48:183–195. 10.1016/j.tranpol.2016.03.007

[CR64] Christoforou Z, Corbillé E, Farhi N, Leurent F (2016) Managing planned disruptions of mass transit systems. Transp Res Rec 2541:46–55. 10.3141/2541-06

[CR65] Chu F, Oetting A (2013) Modeling capacity consumption considering disruption program characteristics and the transition phase to steady operations during disruptions. J Rail Transp Plan Manag 3(3):54–67. 10.1016/j.jrtpm.2013.10.006

[CR66] Corman F, Quaglietta E (2015) Closing the loop in real-time railway control: framework design and impacts on operations. Transp Res Part C Emerg Technol 54:15–39. 10.1016/j.trc.2015.01.014

[CR67] Corman F, D’Ariano A, Pacciarelli D, Pranzo M (2012) Optimal inter-area coordination of train rescheduling decisions. Transp Res Part E Logist Transp Rev 48:71–88. 10.1016/j.tre.2011.05.002

[CR68] Cox A, Prager F, Rose A (2011) Transportation security and the role of resilience: a foundation for operational metrics. Transp Policy 18(2):307–317. 10.1016/j.tranpol.2010.09.004

[CR69] Currie G, Muir C (2017) Understanding passenger perceptions and behaviors during unplanned rail disruptions. Transp Res Procedia 25:4392–4402. 10.1016/j.trpro.2017.05.322

[CR70] Cvetkovic DM, Doob M, Sachs H (1980) Spectra of graphs—theory and application. VEB Deutscher Verlag der Wissenschaften, Berlin

[CR71] Dadfar D, Schwartz F, Voß S (2012) Risk management in global supply chains—hedging for the big bang? In: Mak HY, Lo H (eds) Proceedings of the 17th international conference (HKSTS). vol 2, pp 159–166

[CR72] Daduna J (2020) Evolution of public transport in rural areas—new technologies and digitization. Lect Notes Comput Sci 12202:82–99. 10.1007/978-3-030-49757-6_6

[CR73] Daduna JR, Voß S (1995) Practical experiences in schedule synchronization. Lect Notes Econ Math Syst 430:39–55. 10.1007/978-3-642-57762-8_4

[CR74] Daduna J, Voß S (1996) Efficient technologies for passenger information systems in public mass transit. In: Pirkul H, Shaw M (eds) Proceedings of the first INFORMS conference on information systems and technology. pp 386–391. INFORMS, Washington

[CR75] Daduna J, Voß S (eds) (2000) Informationsmanagement im Verkehr. Physica, Heidelberg. 10.1007/978-3-642-57682-9

[CR76] Daganzo CF (2009) A headway-based approach to eliminate bus bunching: systematic analysis and comparisons. Transp Res Part B Methodol 43:913–921. 10.1016/j.trb.2009.04.002

[CR77] Dakic I, Leclercq L, Menendez M (2021) On the optimization of the bus network design: an analytical approach based on the three-dimensional macroscopic fundamental diagram. Transp Res Part B Methodol 149:393–417. 10.1016/j.trb.2021.04.012

[CR78] D’Ariano A, Meng L, Centulio G, Corman F (2019) Integrated stochastic optimization approaches for tactical scheduling of trains and railway infrastructure maintenance. Comput Ind Eng 127:1315–1335. 10.1016/j.cie.2017.12.010

[CR79] de Oña J, de Oña R, Calvo FJ (2012) A classification tree approach to identify key factors of transit service quality. Expert Syst Appl 39(12):11164–11171. 10.1016/j.eswa.2012.03.037

[CR80] de Souza F, Sebastiani MT (2021) Improving resilience of bus bunching holding strategy through a rolling horizon approach. J Transp Eng Part A Syst 147(10):04021074. 10.1061/JTEPBS.0000587

[CR81] Degeler V, Heydenrijk-Ottens L, Luo D, van Oort N, van Lint H (2021) Unsupervised approach towards analysing the public transport bunching swings formation phenomenon. Public Transp 13:533–555. 10.1007/s12469-020-00251-z

[CR82] Dekker MM, van Lieshout RN, Ball RC, Bouman PC, Dekker SC, Dijkstra HA, Goverde RMP, Huisman D, Panja D, Schaafsma AAM, van den Akker M (2021) A next step in disruption management: combining operations research and complexity science. Public Transp. 10.1007/s12469-021-00261-5

[CR83] Derrible S, Kennedy C (2009) Network analysis of world subway systems using updated graph theory. Transp Res Rec 2112(1):17–25. 10.3141/2112-03

[CR84] Desaulniers G, Hickman MD (2007) Public transit. In: Barnhart C, Laporte G (eds) Transportation. Handbooks in operations research and management science, vol 14. Elsevier, Berlin, pp 69–127. 10.1016/S0927-0507(06)14002-5

[CR85] Dewilde T, Sels P, Cattrysse D, Vansteenwegen P (2013) Robust railway station planning: an interaction between routing, timetabling and platforming. J Rail Transp Plan Manag 3(3):68–77. 10.1016/j.jrtpm.2013.11.002

[CR86] Dimitrov SD, Ceder A (2016) A method of examining the structure and topological properties of public-transport networks. Physica A Stat Mech Appl 451:373–387. 10.1016/j.physa.2016.01.060

[CR87] D’Lima M, Medda F (2015) A new measure of resilience: an application to the London underground. Transp Res Part A Policy Pract 81:35–46. 10.1016/j.tra.2015.05.017

[CR88] Doi T, Nishi T, Voß S (2018) Two-level decomposition-based matheuristic for airline crew rostering problems with fair working time. Eur J Oper Res 267:428–438. 10.1016/j.ejor.2017.11.046

[CR89] Dollevoet T, Huisman D, Schmidt M, Schöbel A (2018) Delay propagation and delay management in transportation networks. In: Borndörfer R, Klug T, Lamorgese L, Mannino C, Reuther M, Schlechte T (eds) Handbook of optimization in the railway industry. Springer, Cham, pp 285–317. 10.1007/978-3-319-72153-8_13

[CR90] Drabicki A, Cats O, Kucharski R (2021) The potential of real-time crowding information in reducing bus bunching under different network saturation levels. In: 7th International conference on models and technologies for intelligent transportation systems (MT-ITS). pp 1–6. 10.1109/MT-ITS49943.2021.9529310

[CR91] Dridi M, Mesghouni K, Borne P (2005) Traffic control in transportation systems. J Manuf Technol Manag 16(1):53–74. 10.1108/17410380510574086

[CR92] Du Q, Kishi K, Aiura N, Nakatsuji T (2014) Transportation network vulnerability: vulnerability scanning methodology applied to multiple logistics transport networks. Transp Res Rec 2410:96–104. 10.3141/2410-11

[CR93] Duarte A, Garcia C, Giannarakis G, Limão S, Polydoropoulou A, Litinas N (2010) New approaches in transportation planning: happiness and transport economics. Netnomics 11:5–32. 10.1007/s11066-009-9037-2

[CR94] Dück V, Ionescu L, Kliewer N, Suhl L (2012) Increasing stability of crew and aircraft schedules. Transp Res Part C Emerg Technol 20(1):47–61. 10.1016/j.trc.2011.02.009

[CR95] Eboli L, Mazzulla G (2007) Service quality attributes affecting customer satisfaction for bus transit. J Public Transp 10(3):21–34. 10.5038/2375-0901.10.3.2

[CR96] Echeverri P, Skålén P (2011) Co-creation and co-destruction: a practice-theory based study of interactive value formation. Market Theory 11(3):351–373. 10.1177/1470593111408181

[CR97] Ehrgott M, Ryan DM (2002) Constructing robust crew schedules with bicriteria optimization. J Multi-Criteria Decis Anal 11(3):139–150. 10.1002/mcda.321

[CR98] Enayatollahi F, Idris AO, Atashgah MAA (2019) Modelling bus bunching under variable transit demand using cellular automata. Public Transp 11:269–298. 10.1007/s12469-019-00203-2

[CR99] European Parliament (2011) Regulation (EU) no 181/2011 of the European Parliament and of the council of 16 February 2011 concerning the rights of passengers in bus and coach transport and amending regulation (EC) no 2006/2004. http://data.europa.eu/eli/reg/2011/181/oj

[CR100] Fan B, Roberts C, Weston P (2012) A comparison of algorithms for minimising delay costs in disturbed railway traffic scenarios. J Rail Transp Plan Manag 2(1):23–33. 10.1016/j.jrtpm.2012.09.002

[CR101] Fang Y, Jiang Y (2019) Replacement service decisions for disruption recovery in light rail systems. Manag Environ Qual 30:286–306. 10.1108/MEQ-08-2017-0074

[CR102] Fang W, Yang S, Yao X (2015) A survey on problem models and solution approaches to rescheduling in railway networks. IEEE Trans Intell Transp Syst 16(6):2997–3016. 10.1109/TITS.2015.2446985

[CR103] Fang Y, Jiang Y, Fei W (2020) Disruption recovery for urban public tram system: an analysis of replacement service selection. IEEE Access 8:31633–31646. 10.1109/ACCESS.2020.2972445

[CR104] Fischetti M, Monaci M (2009) Light robustness. Lect Notes Comput Sci 5868:61–84. 10.1007/978-3-642-05465-5_3

[CR105] Fonzone A, Schmöcker JD, Liu R (2015) A model of bus bunching under reliability-based passenger arrival patterns. Transp Res Procedia 7:276–299. 10.1016/j.trpro.2015.06.015

[CR106] Friedrich M, Müller-Hannemann M, Rückert R, Schiewe A, Schöbel A (2017) Robustness tests for public transport planning. In: D’Angelo G, Dollevoet T (eds) 17th Workshop on algorithmic approaches for transportation modelling, optimization, and systems (ATMOS 2017). Open Access series in informatics (OASIcs), vol 59, pp 6:1–6:16. Schloss Dagstuhl–Leibniz-Zentrum für Informatik, Dagstuhl, Germany. 10.4230/OASIcs.ATMOS.2017.6

[CR107] Friedrich M, Müller-Hannemann M, Rückert R, Schiewe A, Schöbel A (2018) Robustness as a third dimension for evaluating public transport plans. In: Borndörfer R, Storandt S (eds) 18th Workshop on algorithmic approaches for transportation modelling, optimization, and systems (ATMOS 2018). Open Access series in informatics (OASIcs), vol 65, pp 4:1–4:17. Schloss Dagstuhl–Leibniz-Zentrum für Informatik, Dagstuhl, Germany. 10.4230/OASIcs.ATMOS.2018.4

[CR108] Gabrel V, Murat C, Thiele A (2014) Recent advances in robust optimization: an overview. Eur J Oper Res 235:471–483. 10.1016/j.ejor.2013.09.036

[CR109] Gaied M, M’halla A, Lefebvre D, Othmen KB, (2019) Robust control for railway transport networks based on stochastic P-timed Petri net models. Proc Inst Mech Eng Part I J Syst Control Eng 233(7):830–846. 10.1177/0959651818823583

[CR110] Gao Y, Kroon L, Schmidt M, Yang L (2016) Rescheduling a metro line in an over-crowded situation after disruptions. Transp Res Part B Methodol 93:425–449. 10.1016/j.trb.2016.08.011

[CR111] Ge L, Sarhani M, Voß S, Xie L (2021) Review of transit data sources: potentials, challenges and complementarity. Sustainability 13(20):11450. 10.3390/su132011450

[CR112] Ge L, Kliewer N, Nourmohammadzadeh A, Voß S, Xie L (2022) Revisiting the richness of integrated vehicle and crew scheduling. Public Transp. 10.1007/s12469-022-00292-6

[CR113] Gershenson C, Pineda LA (2009) Why does public transport not arrive on time? The pervasiveness of equal headway instability. PLoS ONE 4(10):e7292. 10.1371/journal.pone.0007292 (15 pages) 19862321 10.1371/journal.pone.0007292 PMC2762539

[CR114] Ghaemi N, Cats O, Goverde RM (2018a) Macroscopic multiple-station short-turning model in case of complete railway blockages. Trans Res Part C Emerg Technol 89:113–132. 10.1016/j.trc.2018.02.006

[CR115] Ghaemi N, Zilko AA, Yan F, Cats O, Kurowicka D, Goverde RM (2018b) Impact of railway disruption predictions and rescheduling on passenger delays. J Rail Transp Plan Manag 8(2):103–122. 10.1016/j.jrtpm.2018.02.002

[CR116] Gintner V, Kliewer N, Suhl L (2005) Solving large multiple-depot multiple-vehicle-type bus scheduling problems in practice. OR Spectr 27:507–523. 10.1007/s00291-005-0207-9

[CR117] Gkiotsalitis K, Cats O (2021) At-stop control measures in public transport: literature review and research agenda. Transp Res Part E Logist Transp Rev 145:02176. 10.1016/j.tre.2020.102176

[CR118] Godfrid J, Radnic P, Vaisman A, Zimányi E (2022) Analyzing public transport in the city of Buenos Aires with mobilityDB. Public Transp. 10.1007/s12469-022-00290-8 10.1007/s12469-022-00290-8 PMC8886351 38624856

[CR119] Goerigk M (2015) Exact and heuristic approaches to the robust periodic event scheduling problem. Public Transp 7:101–119. 10.1007/s12469-014-0100-5

[CR120] Goerigk M, Grün B (2014) A robust bus evacuation model with delayed scenario information. OR Spectr 36:923–948. 10.1007/s00291-014-0365-8

[CR121] Golightly D, Dadashi N (2017) The characteristics of railway service disruption: implications for disruption management. Ergonomics 60(3):307–320. 10.1080/00140139.2016.1173231 27215348 10.1080/00140139.2016.1173231

[CR122] Gómez-Ortiz V, Cendales B, Useche S, Bocarejo JP (2018) Relationships of working conditions, health problems and vehicle accidents in bus rapid transit (BRT) drivers. Am J Ind Med 61(4):336–343. 10.1002/ajim.22821 29484691 10.1002/ajim.22821

[CR123] Gong Z, Du B, Liu Z, Zeng W, Perez P, Wu K (2020) SD-seq2seq: a deep learning model for bus bunching prediction based on smart card data. In: 29th International conference on computer communications and networks (ICCCN). pp 1–9. 10.1109/ICCCN49398.2020.9209686

[CR124] Gonzalez-Lopez F, Mejia G, Voß S (2017) Bus rapid transit station CP-net modelling for multi-objective performance evaluation: passenger overcrowding, driving safety, and bus congestion. In: IT/AI for manufacturing (IT), proceedings of the 24th international conference on production research. IFPR, Posnan, Poland

[CR125] Grotenhuis JW, Wiegmans BW, Rietveld P (2007) The desired quality of integrated multimodal travel information in public transport: customer needs for time and effort savings. Transp Policy 14(1):27–38. 10.1016/j.tranpol.2006.07.001

[CR126] Gu W, Yu J, Ji Y, Zheng Y, Zhang HM (2018) Plan-based flexible bus bridging operation strategy. Transp Res Part C Emerg Technol 91:209–229. 10.1016/j.trc.2018.03.015

[CR127] Haghighi N, Liu X, Wei R, Li W, Shao H (2018) Using Twitter data for transit performance assessment: a framework for evaluating transit riders’ opinions about quality of service. Public Transp 10:363–377. 10.1007/s12469-018-0184-4

[CR128] Hartl RF, Hasle G, Janssens GK (2006) Special issue on rich vehicle routing problems. Cent Eur J Oper Res 14(2):103–104. 10.1007/s10100-006-0162-9

[CR129] Hassannayebi E, Sajedinejad A, Mardani S (2016) Disruption management in urban rail transit system: a simulation based optimization approach. In: Handbook of research on emerging innovations in rail transportation engineering, pp 420–450. IGI. 10.4018/978-1-5225-0084-1.ch018

[CR130] Haywood L, Koning M, Monchambert G (2017) Crowding in public transport: who cares and why? Transp Res Part A Policy Pract 100:215–227. 10.1016/j.tra.2017.04.022

[CR131] He SX (2015) An anti-bunching strategy to improve bus schedule and headway reliability by making use of the available accurate information. Comput Ind Eng 85:17–32. 10.1016/j.cie.2015.03.004

[CR132] Heilig L, Voß S (2015) A scientometric analysis of public transport research. J Public Transp 18(2):111–141. 10.5038/2375-0901.18.2.8

[CR133] Heilig L, Negenborn RR, Voß S (2015) Cloud-based intelligent transportation systems using model predictive control. Lect Notes Comput Sci 9335:464–477. 10.1007/978-3-319-24264-4_32

[CR134] Hensher DA, Ho C, Mulley C (2016) Disruption costs in bus contract transitions. Res Transp Econ 59:75–85. 10.1016/j.retrec.2016.04.002

[CR135] Hernández D, Muñoz JC, Giesen R, Delgado F (2015) Analysis of real-time control strategies in a corridor with multiple bus services. Transp Res Part B Methodol 78:83–105. 10.1016/j.trb.2015.04.011

[CR136] Hirschhorn F (2021) A multi-level governance response to the Covid-19 crisis in public transport. Transp Policy 112:13–21. 10.1016/j.tranpol.2021.08.007 10.1016/j.tranpol.2021.08.007 PMC9188819 35719294

[CR137] Hosseini S, Barker K, Ramirez-Marquez JE (2016) A review of definitions and measures of system resilience. Reliab Eng Syst Saf 145:47–61. 10.1016/j.ress.2015.08.006

[CR138] Hosseini S, Ivanov D, Dolgui A (2019) Review of quantitative methods for supply chain resilience analysis. Transp Res Part E Logist Transp Rev 125:285–307. 10.1016/j.tre.2019.03.001

[CR139] Hu H, Gao Y, Yu J, Liu Z, Li X (2016) Planning bus bridging evacuation during rail transit operation disruption. J Urban Plan Dev 142(4):04016015. 10.1061/(ASCE)UP.1943-5444.0000335 (9 pages)

[CR140] Hua W, Ong GP (2018) Effect of information contagion during train service disruption for an integrated rail-bus transit system. Public Transp 10:571–594. 10.1007/s12469-018-0192-4

[CR141] Huang Y, Mannino C, Yang L, Tang T (2020) Coupling time-indexed and big-M formulations for real-time train scheduling during metro service disruptions. Transp Res Part B Methodol 133:38–61. 10.1016/j.trb.2019.12.005

[CR142] Ibarra-Rojas O, Delgado F, Giesen R, Muñoz J (2015) Planning, operation, and control of bus transport systems: a literature review. Transp Res Part B Methodol 77:38–75. 10.1016/j.trb.2015.03.002

[CR143] IEEE (1990) IEEE standard glossary of software engineering terminology. IEEE Std 610.12-1990, pp 1–84. 10.1109/IEEESTD.1990.101064

[CR144] Iliopoulou C, Kepaptsoglou K (2021) Robust electric transit route network design problem (RE-TRNDP) with delay considerations: model and application. Transp Res Part C Emerg Technol 129:103255. 10.1016/j.trc.2021.103255

[CR145] Iliopoulou CA, Milioti CP, Vlahogianni EI, Kepaptsoglou KL (2020) Identifying spatio-temporal patterns of bus bunching in urban networks. J Intell Transp Syst 24:365–382. 10.1080/15472450.2020.1722949

[CR146] Ingels J, Maenhout B (2015) The impact of reserve duties on the robustness of a personnel shift roster: an empirical investigation. Comput Oper Res 61:153–169. 10.1016/j.cor.2015.03.010

[CR147] Ionescu L (2018) Robust Efficiency of Airline Resource Schedules. Ph.D. thesis, Fachbereich Wirtschaftswissenschaft, Freie Universität Berlin

[CR148] Ionescu L, Kliewer N (2011) Increasing flexibility of airline crew schedules. Procedia Soc Behav Sci 20:1019–1028. 10.1016/j.sbspro.2011.08.111

[CR149] Jamili A, Pourseyed Aghaee M (2015) Robust stop-skipping patterns in urban railway operations under traffic alteration situation. Transp Res Part C Emerg Technol 61:63–74. 10.1016/j.trc.2015.09.013

[CR150] Janarthanan N, Schneider JB (1984) Computer-aided design as applied to transit system emergency contingency planning. Comput Environ Urban Syst 9(1):33–52. 10.1016/0198-9715(84)90004-8

[CR151] Jara-Díaz S, Gschwender A (2003) Towards a general microeconomic model for the operation of public transport. Transp Rev 23(4):453–469. 10.1080/0144164032000048922

[CR152] Jenelius E (2010) Redundancy importance: links as rerouting alternatives during road network disruptions. Procedia Eng 3:129–137. 10.1016/j.proeng.2010.07.013

[CR153] Jenelius E, Cats O (2015) The value of new public transport links for network robustness and redundancy. Transportmetrica A Transp Sci 11(9):819–835. 10.1080/23249935.2015.1087232

[CR154] Jevinger Å, Persson JA (2019) Exploring the potential of using real-time traveler data in public transport disturbance management. Public Transp 11(2):413–441. 10.1007/s12469-019-00209-w

[CR155] Jevinger Å, Persson JA (2020) Disturbance management and information availability in public transport, with focus on Scania County, Sweden. In: Bougdah H, Versaci A, Sotoca A, Trapani F, Migliore M, Clark N (eds) Urban and transit planning: a culmination of selected research papers from IEREK conferences on urban planning, architecture and green urbanism, Italy and Netherlands (2017). Springer, Cham. pp 305–311. 10.1007/978-3-030-17308-1_29

[CR156] Jiang F, Cacchiani V, Toth P (2017) Train timetabling by skip-stop planning in highly congested lines. Transp Res Part B Methodol 104:149–174. 10.1016/j.trb.2017.06.018

[CR157] Jin JG, Tang LC, Sun L, Lee DH (2014) Enhancing metro network resilience via localized integration with bus services. Transp Res Part E Logist Transp Rev 63:17–30. 10.1016/j.tre.2014.01.002

[CR158] Jin JG, Teo KM, Odoni AR (2016) Optimizing bus bridging services in response to disruptions of urban transit rail networks. Transp Sci 50(3):790–804. 10.1287/trsc.2014.0577

[CR159] Jovanović P, Kecman P, Bojović N, Mandić D (2017) Optimal allocation of buffer times to increase train schedule robustness. Eur J Oper Res 256:44–54. 10.1016/j.ejor.2016.05.013

[CR160] Jovanovic R, Tuba M, Voß S (2019) Fixed set search applied to the traveling salesman problem. Lect Notes Comput Sci 11299:63–77. 10.1007/978-3-030-05983-5_5

[CR161] Kang L, Wu J, Sun H, Zhu X, Wang B (2015) A practical model for last train rescheduling with train delay in urban railway transit networks. Omega 50:29–42. 10.1016/j.omega.2014.07.005

[CR162] Kang L, Zhu X, Sun H, Wu J, Gao Z, Hu B (2019) Last train timetabling optimization and bus bridging service management in urban railway transit networks. Omega 84:31–44. 10.1016/j.omega.2018.04.003

[CR163] Kang L, Meng Q, Zhou C (2020) Bus driver scheduling enhancement: a derandomizing approach for uncertain bus trip times. Transp B Transp Dyn 8(1):200–218. 10.1080/21680566.2019.1695153

[CR164] Karl A (2018) Commercial services in German local public transport. Res Transp Econ 69:319–325. 10.1016/j.retrec.2018.03.004

[CR165] Kepaptsoglou K, Karlaftis MG (2009) The bus bridging problem in metro operations: conceptual framework, models and algorithms. Public Transp 1(4):275–297. 10.1007/s12469-010-0017-6

[CR166] Khoshniyat F, Peterson A (2017) Improving train service reliability by applying an effective timetable robustness strategy. J Intell Transp Syst 21:525–543. 10.1080/15472450.2017.1326114

[CR167] Kiefer A, Kritzinger S, Doerner K (2016) Disruption management for the Viennese public transport provider. Public Transp 8:161–183. 10.1007/s12469-016-0123-1

[CR168] Kiefer A, Schilde M, Doerner KF (2018) Scheduling of maintenance work of a large-scale tramway network. Eur J Oper Res 270:1158–1170. 10.1016/j.ejor.2018.04.027

[CR169] Kindlmann P, Burel F (2008) Connectivity measures: a review. Landsc Ecol 23:879–890. 10.1007/s10980-008-9245-4

[CR170] Knoop VL, Snelder M, van Zuylen HJ, Hoogendoorn SP (2012) Link-level vulnerability indicators for real-world networks. Transp Res Part A Policy Pract 46:843–854. 10.1016/j.tra.2012.02.004

[CR171] Kokkinogenis Z, Filguieras J, Carvalho S, Sarmento L, Rossetti RJ (2015) Mobility network evaluation in the user perspective: real-time sensing of traffic information in Twitter messages. In: Rossetti RJ, Liu R (eds) Advances in artificial transportation systems and simulation. Academic Press, Boston, pp 219–234. 10.1016/B978-0-12-397041-1.00012-1

[CR172] König E (2020) A review on railway delay management. Public Transp 12:335–361. 10.1007/s12469-020-00233-1

[CR173] Kroon L, Huisman D (2011) Algorithmic support for railway disruption management. In: van Nunen J, Huijbregts P, Rietveld P (eds) Transitions towards sustainable mobility. Springer, Berlin, pp 193–210. 10.1007/978-3-642-21192-8_11

[CR174] Lai DSW, Leung JMY (2018) Real-time rescheduling and disruption management for public transit. Transportmetrica B Transp Dyn 6(1):17–33. 10.1080/21680566.2017.1358678

[CR175] Larsen R, Pranzo M, D’Ariano A, Corman F, Pacciarelli D (2014) Susceptibility of optimal train schedules to stochastic disturbances of process times. Flex Serv Manuf J 26:466–489. 10.1007/s10696-013-9172-9

[CR176] Lee Y, Lu LS, Wu ML, Lin DY (2017) Balance of efficiency and robustness in passenger railway timetables. Transp Res Part B Methodol 97:142–156. 10.1016/j.trb.2016.12.004

[CR177] Leng N, Corman F (2020) The role of information availability to passengers in public transport disruptions: an agent-based simulation approach. Transp Res Part A Policy Pract 133:214–236. 10.1016/j.tra.2020.01.007

[CR178] Li S, Liu R, Yang L, Gao Z (2019) Robust dynamic bus controls considering delay disturbances and passenger demand uncertainty. Transp Res Part B Methodol 123:88–109. 10.1016/j.trb.2019.03.019

[CR179] Liang J, Wu J, Qu Y, Yin H, Qu X, Gao Z (2019) Robust bus bridging service design under rail transit system disruptions. Transp Res Part E Logist Transp Rev 132:97–116. 10.1016/j.tre.2019.10.008

[CR180] Liao F, van Wee B (2017) Accessibility measures for robustness of the transport system. Transportation 44:1213–1233. 10.1007/s11116-016-9701-y

[CR181] Lidén T (2020) Coordinating maintenance windows and train traffic: a case study. Public Transp 12:261–298. 10.1007/s12469-020-00232-2

[CR182] Lidén T, Kalinowski T, Waterer H (2018) Resource considerations for integrated planning of railway traffic and maintenance windows. J Rail Transp Plan Manag 8(1):1–15. 10.1016/j.jrtpm.2018.02.001

[CR183] Liebchen C, Lübbecke M, Möhring R, Stiller S (2009) The concept of recoverable robustness, linear programming recovery, and railway applications. Lect Notes Comput Sci 5868:1–27. 10.1007/978-3-642-05465-5_1

[CR184] Liebchen C, Schachtebeck M, Schöbel A, Stiller S, Prigge A (2010) Computing delay resistant railway timetables. Comput Oper Res 37(5):857–868. 10.1016/j.cor.2009.03.022

[CR185] Lin DY, Juan CJ, Chang CC (2020) A branch-and-price-and-cut algorithm for the integrated scheduling and rostering problem of bus drivers. J Adv Transp. 10.1155/2020/3153201 (Article ID 3153201)

[CR186] Ling X, Peng Y, Sun S, Li P, Wang P (2018) Uncovering correlation between train delay and train exposure to bad weather. Physica A Stat Mech Appl 512:1152–1159. 10.1016/j.physa.2018.07.057

[CR187] Liu R, Palm M, Shalaby A, Farber S (2020) A social equity lens on bus bridging and ride-hailing responses to unplanned subway disruptions. J Transp Geogr 88:102870. 10.1016/j.jtrangeo.2020.102870

[CR188] Louwerse I, Huisman D (2014) Adjusting a railway timetable in case of partial or complete blockades. Eur J Oper Res 235:583–593. 10.1016/j.ejor.2013.12.020

[CR190] Luo C, Li X, Zhou Y, Caunhye AM, Alibrandi U, Aydin NY, Ratti C, Eckhoff D, Bojic I (2019) Data-driven disruption response planning for a mass rapid transit system. In: Qu X, Zhen L, Howlett RJ, Jain LC (eds) Smart transportation systems 2019. Springer, Singapore, pp 205–213. 10.1007/978-981-13-8683-1_21

[CR189] Luo C, Xu L (2021) Railway disruption management: designing bus bridging services under uncertainty. Comput Oper Res 131:105284. 10.1016/j.cor.2021.105284

[CR191] Lusby RM, Larsen J, Bull S (2018) A survey on robustness in railway planning. Eur J Oper Res 266:1–15. 10.1016/j.ejor.2017.07.044

[CR192] Maas C (1987) Transportation in graphs and the admittance spectrum. Discret Appl Math 16(1):31–49. 10.1016/0166-218X(87)90052-7

[CR193] Maenhout B, Vanhoucke M (2018) A perturbation matheuristic for the integrated personnel shift and task re-scheduling problem. Eur J Oper Res 269:806–823. 10.1016/j.ejor.2018.03.005

[CR194] Mahdavi SMH, Bhouri N, Scemama G (2020) Dynamic resilience of public transport network: a case study for fleet-failure in bus transport operation of New Delhi. Transp Res Procedia 47:672–679. 10.1016/j.trpro.2020.03.146

[CR195] Malandri C, Fonzone A, Cats O (2018) Recovery time and propagation effects of passenger transport disruptions. Physica A Stat Mech Appl 505:7–17. 10.1016/j.physa.2018.03.028

[CR196] Malucelli F, Tresoldi E (2019) Delay and disruption management in local public transportation via real-time vehicle and crew re-scheduling: a case study. Public Transp 11(1):1–25. 10.1007/s12469-019-00196-y

[CR197] Maniezzo V, Stützle T, Voß S (eds) (2009) Matheuristics: hybridizing metaheuristics and mathematical programming. Springer, Berlin. 10.1007/978-1-4419-1306-7

[CR198] Marsden G, Docherty I (2021) Mega-disruptions and policy change: lessons from the mobility sector in response to the Covid-19 pandemic in the UK. Transp Policy 110:86–97. 10.1016/j.tranpol.2021.05.015 10.1016/j.tranpol.2021.05.015 PMC9761561 36567697

[CR199] Mattsson LG, Jenelius E (2015) Vulnerability and resilience of transport systems—a discussion of recent research. Transp Res Part A Policy Pract 81:16–34. 10.1016/j.tra.2015.06.002

[CR200] Mesquita M, Paias A, Respício A (2009) Branching approaches for integrated vehicle and crew scheduling. Public Transp 1:21–37. 10.1007/s12469-008-0005-2

[CR201] Mesquita M, Moz M, Paias A, Pato M (2013) A decomposition approach for the integrated vehicle-crew-roster problem with days-off pattern. Eur J Oper Res 229:318–331. 10.1016/j.ejor.2013.02.055

[CR202] Mhalla A, Gaied M (2018) Modeling and robustness study of railway transport networks using P-timed Petri nets. J Eng. 10.1155/2018/2083576 (Article ID 2083576)

[CR203] Mishra S, Welch TF, Jha MK (2012) Performance indicators for public transit connectivity in multi-modal transportation networks. Transp Res Part A Policy Pract 46:1066–1085. 10.1016/j.tra.2012.04.006

[CR204] Molenbruch Y, Braekers K, Caris A (2017) Typology and literature review for dial-a-ride problems. Ann Oper Res 259:295–325. 10.1007/s10479-017-2525-0

[CR205] Monchambert G, de Palma A (2014) Public transport reliability and commuter strategy. J Urban Econ 81:14–29. 10.1016/j.jue.2014.02.001

[CR206] Moreira-Matias L, Cats O, Gama J, Mendes-Moreira J, de Sousa JF (2016) An online learning approach to eliminate bus bunching in real-time. Appl Soft Comput 47:460–482. 10.1016/j.asoc.2016.06.031

[CR207] Mouronte-López ML (2021) Analysing the vulnerability of public transport networks. J Adv Transp 2021:5513311. 10.1155/2021/5513311

[CR208] Mouwen A (2015) Drivers of customer satisfaction with public transport services. Transp Res Part A Policy Pract 78:1–20. 10.1016/j.tra.2015.05.005

[CR209] Mudigonda S, Ozbay K, Bartin B (2019) Evaluating the resilience and recovery of public transit system using big data: case study from New Jersey. J Transp Saf Secur 11(5):491–519. 10.1080/19439962.2018.1436105

[CR210] Mützel CM, Scheiner J (2021) Investigating spatio-temporal mobility patterns and changes in metro usage under the impact of COVID-19 using Taipei metro smart card data. Public Transp. 10.1007/s12469-021-00280-2 10.1007/s12469-021-00280-2 PMC8365295 38624766

[CR211] Nabais JL, Negenborn RR, Botto MA (2012) A novel predictive control based framework for optimizing intermodal container terminal operations. Lect Notes Comput Sci 7555:53–71. 10.1007/978-3-642-33587-7_4

[CR212] National Academies of Sciences, Engineering, and Medicine (2013) A transportation guide for all-hazards emergency evacuation. The National Academies Press, Washington, DC. Final research report. 10.17226/22586

[CR213] National Academies of Sciences, Engineering, and Medicine (ed) (2015) Open data: challenges and opportunities for transit agencies. The National Academies Press, Washington, DC. 10.17226/22195

[CR214] Nesheli M, Ceder A (2014) Optimal combinations of selected tactics for public-transport transfer synchronization. Transp Res Part C Emerg Technol 48:491–504. 10.1016/j.trc.2014.09.013

[CR215] Nesheli M, Ceder A (2015) Improved reliability of public transportation using real-time transfer synchronization. Transp Res Part C Emerg Technol 60:525–539. 10.1016/j.trc.2015.10.006

[CR216] Nesheli MM, Ceder AA, Brissaud R (2017) Public transport service-quality elements based on real-time operational tactics. Transportation 44:957–975. 10.1007/s11116-016-9688-4

[CR217] Newton A, Johnson S, Bowers K (2004) Crime on bus routes: an evaluation of a safer travel initiative. Polic Int J 27(3):302–319. 10.1108/13639510410553086

[CR218] Ng M, Lo HK (2016) Robust models for transportation service network design. Transp Res Part B Methodol 94:378–386. 10.1016/j.trb.2016.10.001

[CR219] Nguyen P, Diab E, Shalaby A (2019a) Understanding the factors that influence the probability and time to streetcar bunching incidents. Public Transp 11:299–320. 10.1007/s12469-019-00201-4

[CR220] Nguyen T, Xie M, Liu X, Arunachalam N, Rau A, Lechner B, Busch F, Wong Y (2019b) Platooning of autonomous public transport vehicles: the influence of ride comfort on travel delay. Sustainability 11(19):1–14. 10.3390/su11195237 (paper 5237)

[CR221] Nguyen-Phuoc D, Young W, Currie G, De Gruyter C (2020) Traffic congestion relief associated with public transport—state-of-the-art. Public Transp 12:455–481. 10.1007/s12469-020-00231-3

[CR222] Nian G, Chen F, Li Z, Zhu Y, Sun DJ (2019) Evaluating the alignment of new metro line considering network vulnerability with passenger ridership. Transportmetrica A Transp Sci 15:1402–1418. 10.1080/23249935.2019.1599080

[CR223] Nicholson A, Schmöcker J, Bell M, Iida Y (2003) Assessing transport reliability: malevolence and user knowledge. In: Bell M, Iida Y (eds) The network reliability of transport. Emerald, Bingley, pp 1–22. 10.1108/9781786359544-001

[CR224] Nimpanomprasert T, Xie L, Kliewer N (2022) Comparing two hybrid neural network models to predict real-world bus travel time. Transp Res Procedia 62:393–400. 10.1016/j.trpro.2022.02.049

[CR225] Økland A, Olsson NO (2021) Punctuality development and delay explanation factors on Norwegian railways in the period 2005–2014. Public Transp 13:127–161

[CR226] Pahl J, Voß S, Woodruff DL (2007) Production planning with load dependent lead times: an update of research. Ann Oper Res 153:297–345. 10.1007/s10479-007-0173-5

[CR227] Pahl J, Voß S (2022) How to get it right: structured literature reviews in engineering and management sciences. Tech. rep., Department of Technology and Innovation, University of Southern Denmark, Odense and Institute of Information Systems (IWI), University of Hamburg

[CR228] Papangelis K, Velaga NR, Ashmore F, Sripada S, Nelson JD, Beecroft M (2016) Exploring the rural passenger experience, information needs and decision making during public transport disruption. Res Transp Bus Manag 18:57–69. 10.1016/j.rtbm.2016.01.002

[CR229] Parbo J, Nielsen OA, Prato CG (2016) Passenger perspectives in railway timetabling: a literature review. Transp Rev 36(4):500–526. 10.1080/01441647.2015.1113574

[CR230] Parragh SN, Doerner KF, Hartl RF (2008) A survey on pickup and delivery problems. Journal für Betriebswirtschaft 58(2):81–117. 10.1007/s11301-008-0036-4

[CR231] Peled I, Lee K, Jiang Y, Dauwels J, Pereira FC (2019) Online predictive optimization framework for stochastic demand-responsive transit services. Tech. rep., Technical University of Denmark (DTU), Kgs. Lyngby, Denmark. https://arxiv.org/pdf/1902.09745.pdf

[CR232] Pellegrini P, Marlière G, Rodriguez J (2017) RECIFE-SAT: a MILP-based algorithm for the railway saturation problem. J Rail Transp Plan Manag 7(1):19–32. 10.1016/j.jrtpm.2017.08.001

[CR233] Pender B, Currie G, Delbosc A, Shiwakoti N (2013) Disruption recovery in passenger railways: international survey. Transp Res Rec 2353(1):22–32. 10.3141/2353-03

[CR234] Pender B, Currie G, Delbosc A, Shiwakoti N (2014a) Improving bus bridging responses via satellite bus reserve locations. J Transp Geogr 34:202–210. 10.1016/j.jtrangeo.2013.12.007

[CR235] Pender B, Currie G, Delbosc A, Shiwakoti N (2014b) Social media use during unplanned transit network disruptions: a review of literature. Transp Rev 34(4):501–521. 10.1080/01441647.2014.915442

[CR236] Pender B, Currie G, Shiwakoti N, Delbosc A (2015) Economic viability of bus bridging reserves for fast response to unplanned passenger rail disruption. Transp Res Rec 2537(1):13–22. 10.3141/2537-02

[CR237] Petit A, Lei C, Ouyang Y (2019) Multiline bus bunching control via vehicle substitution. Transp Res Part B Methodol 126:68–86. 10.1016/j.trb.2019.05.009

[CR238] Piner D, Condry B (2017) International best practices in managing unplanned disruption to suburban rail services. Transp Res Procedia 25:4403–4410. 10.1016/j.trpro.2017.05.331

[CR239] Potthoff D, Huisman D, Desaulniers G (2010) Column generation with dynamic duty selection for railway crew rescheduling. Transp Sci 44:493–505. 10.1287/trsc.1100.0322

[CR240] Qi J, Cacchiani V, Yang L (2018) Robust train timetabling and stop planning with uncertain passenger demand. Electron Notes Discret Math 69:213–220. 10.1016/j.endm.2018.07.028

[CR241] Rahimi Siegrist M, Corman F (2021) Modeling and quantifying interaction of information and capacity in public transport disruptions. J Adv Transp 2021:5398316. 10.1155/2021/5398316

[CR242] Rahimi E, Shamshiripour A, Shabanpour R, Mohammadian A, Auld J (2019) Analysis of transit users’ waiting tolerance in response to unplanned service disruptions. Transp Res Part D Transp Environ 77:639–653. 10.1016/j.trd.2019.10.011

[CR243] Redmond M, Campbell A, Ehmke J (2020) Data-driven planning of reliable itineraries in multi-modal transit networks. Public Transp 12:171–205. 10.1007/s12469-019-00221-0

[CR244] Reggiani A (2013) Network resilience for transport security: some methodological considerations. Transp Policy 28:63–68. 10.1016/j.tranpol.2012.09.007

[CR245] Reggiani A, Nijkamp P, Lanzi D (2015) Transport resilience and vulnerability: the role of connectivity. Transp Res Part A Policy Pract 81:4–15. 10.1016/j.tra.2014.12.012

[CR246] Ren G, He Y, Yu Z, Ouyang Y, Xu L (2019) Resilience enhancing strategy and model of compound public transit network based on disruption situation. In: CICTP 2019. pp 3464–3475. 10.1061/9780784482292.300

[CR247] Rezanova NJ, Ryan DM (2010) The train driver recovery problem—a set partitioning based model and solution method. Comput Oper Res 37:845–856. 10.1016/j.cor.2009.03.023

[CR248] Riener A, Appel A, Dorner W, Huber T, Kolb JC, Wagner H (eds) (2020) Autonome Shuttlebusse im ÖPNV. Springer, Berlin. 10.1007/978-3-662-59406-3

[CR249] Rietveld P, Bruinsma F, van Vuuren D (2001) Coping with unreliability in public transport chains: a case study for Netherlands. Transp Res Part A Policy Pract 35(6):539–559. 10.1016/S0965-8564(00)00006-9

[CR250] Risser R, Lexell E, Bell D, Iwarsson S, Ståhl A (2015) Use of local public transport among people with cognitive impairments—a literature review. Transp Res Part F Traff Psychol Behav 29:83–97. 10.1016/j.trf.2015.01.002

[CR251] Sajikumar S, Bijulal D (2021) Zero bunching solution for a local public transport system with multiple-origins bus operation. Public Transp. 10.1007/s12469-021-00273-1

[CR252] Sarhani M, Voß S (2022) Prediction of transit delays with machine learning: how to exploit open data sources. Institute of Information Systems (IWI), University of Hamburg, Tech. rep

[CR253] Saw VL, Chung NN, Quek WL, Pang YEI, Chew LY (2019) Bus bunching as a synchronisation phenomenon. Sci Rep 9(1):6887. 10.1038/s41598-019-43310-7 31053731 10.1038/s41598-019-43310-7 PMC6499774

[CR254] Saxena N, Hossein Rashidi T, Auld J (2019) Studying the tastes effecting mode choice behavior of travelers under transit service disruptions. Travel Behav Soc 17:86–95. 10.1016/j.tbs.2019.07.004

[CR255] Schmidt M (2014) Integrating routing decisions in public transportation problems. Springer, New York. 10.1007/978-1-4614-9566-6

[CR256] Schmittner C, Tummeltshammer P, Hofbauer D, Shaaban AM, Meidlinger M, Tauber M, Bonitz A, Hametner R, Brandstetter M (2019) Threat modeling in the railway domain. Lect Notes Comput Sci 11495:261–271. 10.1007/978-3-030-18744-6_17

[CR257] Schmöcker JD, Sun W, Fonzone A, Liu R (2016) Bus bunching along a corridor served by two lines. Transp Res Part B Methodol 93:300–317. 10.1016/j.trb.2016.07.005

[CR258] Schöbel A (2006) Optimization in public transportation. Springer, New York. 10.1007/978-0-387-36643-2

[CR259] Schöbel A (2012) Line planning in public transportation: models and methods. OR Spectr 34:491–510. 10.1007/s00291-011-0251-6

[CR260] Schöbel A (2014) Generalized light robustness and the trade-off between robustness and nominal quality. Math Methods Oper Res 80(2):161–191. 10.1007/s00186-014-0474-9

[CR261] Schöbel A, Pätzold J, Müller JP (2019) The trickle-in effect: modeling passenger behavior in delay management. In: Cacchiani V, Marchetti-Spaccamela A (eds) 19th Symposium on algorithmic approaches for transportation modelling, optimization, and systems (ATMOS 2019). Open Access series in informatics (OASIcs), vol 75, pp 6:1–6:15. Schloss Dagstuhl–Leibniz-Zentrum für Informatik. 10.4230/OASIcs.ATMOS.2019.6

[CR262] Scholz G (2012) IT-Systeme für Verkehrsunternehmen. dpunkt, Heidelberg

[CR263] Sethuraman G, Liu X, Bachmann FR, Xie M, Ongel A, Busch F (2019) Effects of bus platooning in an urban environment. In: IEEE intelligent transportation systems conference (ITSC). pp 974–980. 10.1109/ITSC.2019.8917041

[CR264] Shakibayifar M, Sheikholeslami A, Corman F (2018) A simulation-based optimization approach to reschedule train traffic in uncertain conditions during disruptions. Sci Iran 25(2):646–662. 10.24200/sci.2017.4186

[CR265] Shalaby A, Li L, Diab E (2021) Rail transit disruption management: a comprehensive review of strategies and approaches. In: Currie G (ed) Handbook of public transport research. Edward Elgar, Cheltenham, pp 280–313. 10.4337/9781788978668.00022

[CR266] Shi X, Voß S (2007) Container terminal operations under the influence of shipping alliances. In: Bichou K, Bell M, Evans A (eds) Risk management in port operations, logistics and supply chain security. Informa, London, pp 135–164. 10.4324/9781315850504

[CR267] Shires J, Ojeda-Cabral M, Wardman M (2019) The impact of planned disruptions on rail passenger demand. Transportation 46:1807–1837. 10.1007/s11116-018-9889-0

[CR268] Simons R (2019) The influence of railway signalling characteristics on resilience. Master thesis, Transport, Infrastructure and Logistics, Delft University of Technology. https://repository.tudelft.nl/islandora/object/uuid:0e76a919-3d02-4282-a56b-dd20a94b61fa/datastream/OBJ/download

[CR269] Sodhi M, Tang C (2012) Managing supply chain risk. Springer, New York. 10.1007/978-1-4614-3238-8

[CR270] Solinen E, Nicholson G, Peterson A (2017) A microscopic evaluation of railway timetable robustness and critical points. J Rail Transp Plan Manag 7(4):207–223. 10.1016/j.jrtpm.2017.08.005

[CR271] Soza-Parra J, Raveau S, Muñoz J, Cats O (2019) The underlying effect of public transport reliability on users’ satisfaction. Transp Res Part A Policy Pract 126:83–93. 10.1016/j.tra.2019.06.004

[CR272] Sparing D, Goverde R (2013) Identifying effective guaranteed connections in a multimodal public transport network. Public Transp 5:79–94. 10.1007/s12469-013-0068-6

[CR273] Su Z, Jamshidi A, Núñez A, Baldi S, de Schutter B (2017) Multi-level condition-based maintenance planning for railway infrastructures—a scenario-based chance-constrained approach. Transp Res Part C Emerg Technol 84:92–123. 10.1016/j.trc.2017.08.018

[CR274] Sugishita K, Asakura Y (2020) Citation network analysis of vulnerability studies in the fields of transportation and complex networks. Transp Res Procedia 47:369–376. 10.1016/j.trpro.2020.03.111

[CR275] Sugishita K, Asakura Y (2021) Vulnerability studies in the fields of transportation and complex networks: a citation network analysis. Public Transp 13:1–34. 10.1007/s12469-020-00247-9

[CR276] Sun DJ, Guan S (2016) Measuring vulnerability of urban metro network from line operation perspective. Transp Res Part A Policy Pract 94:348–359. 10.1016/j.tra.2016.09.024

[CR277] Sun W, Schmöcker JD (2018) Considering passenger choices and overtaking in the bus bunching problem. Transportmetrica B Transp Dyn 6:151–168. 10.1080/21680566.2017.1387876

[CR278] Sun W, Schmöcker JD, Nakamura T (2021) On the tradeoff between sensitivity and specificity in bus bunching prediction. J Intell Transp Syst 25:384–400. 10.1080/15472450.2020.1725887

[CR279] Szymański P, Żołnieruk M, Oleszczyk P, Gisterek I, Kajdanowicz T (2018) Spatio-temporal profiling of public transport delays based on large-scale vehicle positioning data from GPS in Wrocław. IEEE Trans Intell Transp Syst 19(11):3652–3661. 10.1109/TITS.2018.2852845

[CR280] Tahmasseby S, van Nes R (2007) Robustness of urban public transport networks. WIT Trans Built Environ 96:337–346. 10.2495/UT070321

[CR281] Tang X, Lin X, He F (2019) Robust scheduling strategies of electric buses under stochastic traffic conditions. Transp Res Part C Emerg Technol 105:163–182. 10.1016/j.trc.2019.05.032

[CR282] Tang J, Xu L, Luo C, Ng TSA (2021) Multi-disruption resilience assessment of rail transit systems with optimized commuter flows. Reliab Eng Syst Saf 214:107715. 10.1016/j.ress.2021.107715

[CR283] Taylor MAP (2017) Vulnerability analysis for transportation networks. Elsevier, Amsterdam

[CR284] Thomas L, Rhind D, Robinson K (2006) Rail passenger perceptions of risk and safety and priorities for improvement. Cognit Technol Work 8:67–75. 10.1007/s10111-005-0021-9

[CR285] Tian S (2021) A short-turning strategy for the management of bus bunching considering variable spatial-temporal running time. J Uncertain Syst 14(3):2150020. 10.1142/S1752890921500203

[CR286] Tian S, Li X, Liu J, Ma H, Yu H (2022) A short-turning strategy to alleviate bus bunching. J Amb Intell Human Comput 13(1):117–128. 10.1007/s12652-020-02891-2

[CR287] Tirachini A, Cortés C, Jara-Díaz S (2011) Optimal design and benefits of a short turning strategy for a bus corridor. Transportation 38:169–189. 10.1007/s11116-010-9287-8

[CR288] TRB (2008) The role of transit in emergency evacuation. Transportation Research Board, Washington, DC. Special Report 294. http://onlinepubs.trb.org/onlinepubs/sr/sr294.pdf

[CR289] van der Hurk E, Koutsopoulos HN, Wilson N, Kroon LG, Maróti G (2016) Shuttle planning for link closures in urban public transport networks. Transp Sci 50:947–965. 10.1287/trsc.2015.0647

[CR290] van Exel N, Rietveld P (2009) When strike comes to town... anticipated and actual behavioural reactions to a one-day, pre-announced, complete rail strike in the Netherlands. Transp Res Part A Policy Pract 43(5):526–535. 10.1016/j.tra.2009.01.003

[CR291] van Kooten Niekerk M (2018) Optimizing for reliable and sustainable public transport. Ph.D. thesis, Universiteit Utrecht, Utrecht

[CR292] van Lierop D, Badami MG, El-Geneidy AM (2018) What influences satisfaction and loyalty in public transport? A review of the literature. Transp Rev 38(1):52–72. 10.1080/01441647.2017.1298683

[CR293] van Oort N (2021) Service reliability: a planning and operations perspective. In: Currie G (ed) Handbook of public transport research. Edward Elgar, Cheltenham, pp 252–279. 10.4337/9781788978668.00021

[CR294] Vansteenwegen P, Dewilde T, Burggraeve S, Cattrysse D (2016) An iterative approach for reducing the impact of infrastructure maintenance on the performance of railway systems. Eur J Oper Res 252:39–53. 10.1016/j.ejor.2015.12.037

[CR295] Varga B, Tettamanti T, Kulcsár B (2019) Energy-aware predictive control for electrified bus networks. Appl Energy. 10.1016/j.apenergy.2019.113477 (Article 113477)

[CR296] Veelenturf LP, Potthoff D, Huisman D, Kroon LG (2012) Railway crew rescheduling with retiming. Transp Res Part C Emerg Technol 20(1):95–110. 10.1016/j.trc.2010.09.008

[CR297] Veelenturf LP, Kidd MP, Cacchiani V, Kroon LG, Toth P (2016a) A railway timetable rescheduling approach for handling large-scale disruptions. Transp Sci 50:841–862. 10.1287/trsc.2015.0618

[CR298] Veelenturf LP, Potthoff D, Huisman D, Kroon LG, Maróti G, Wagelmans AP (2016b) A quasi-robust optimization approach for crew rescheduling. Transp Sci 50:204–215. 10.1287/trsc.2014.0545

[CR299] Vepsäläinen J, Kivekäs K, Otto K, Lajunen A, Tammi K (2018) Development and validation of energy demand uncertainty model for electric city buses. Transp Res Part D Transp Environ 63:347–361. 10.1016/j.trd.2018.06.004

[CR300] Vepsäläinen J, Otto K, Lajunen A, Tammi K (2019) Computationally efficient model for energy demand prediction of electric city bus in varying operating conditions. Energy 169:433–443. 10.1016/j.energy.2018.12.064

[CR301] Verbich D, Diab E, El-Geneidy A (2016) Have they bunched yet? An exploratory study of the impacts of bus bunching on dwell and running times. Public Transp 8:225–242. 10.1007/s12469-016-0126-y

[CR302] Verhaegh T, Huisman D, Fioole P, Vera JC (2017) A heuristic for real-time crew rescheduling during small disruptions. Public Transp 9:325–342. 10.1007/s12469-017-0155-1

[CR303] Vickrey WS (1955) A proposal for revising New York’s subway fare structure. J Oper Res Soc Am 3(1):38–68. 10.1287/opre.3.1.38

[CR304] Vickrey WS (1963) Pricing in urban and suburban transport. Am Econ Rev 53(2):452–465

[CR305] Vodopivec N, Miller-Hooks E (2019) Transit system resilience: quantifying the impacts of disruptions on diverse populations. Reliab Eng Syst Saf. 10.1016/j.ress.2019.106561 (paper 106561)

[CR306] Voß S (1992) Network design formulations in schedule synchronization. Lect Notes Econ Math Syst 386:137–152. 10.1007/978-3-642-85968-7_10

[CR307] Voß S, Gutenschwager K (2001) Informationsmanagement. Springer, Berlin. 10.1007/978-3-642-56878-7

[CR308] Voß S, Martello S, Osman IH, Roucairol C (eds) (1999) Meta-heuristics: advances and trends in local search paradigms for optimization. Kluwer, Boston. 10.1007/978-1-4615-5775-3

[CR309] Voß S, Mejia G, Voß A (2020) Mystery shopping in public transport: the case of bus station design. Lect Notes Comput Sci 12423:527–542. 10.1007/978-3-030-60114-0_36

[CR310] Vromans MJ, Dekker R, Kroon LG (2006) Reliability and heterogeneity of railway services. Eur J Oper Res 172:647–665. 10.1016/j.ejor.2004.10.010

[CR311] Vuchic VR (2005) Urban transit: operations, planning, and economics. Wiley, Hoboken

[CR312] Wan C, Yang Z, Zhang D, Yan X, Fan S (2018) Resilience in transportation systems: a systematic review and future directions. Transp Rev 38(4):479–498. 10.1080/01441647.2017.1383532

[CR313] Wang J, Sun L (2020) Dynamic holding control to avoid bus bunching: a multi-agent deep reinforcement learning framework. Transp Res Part C Emerg Technol 116:102661. 10.1016/j.trc.2020.102661

[CR314] Wang Y, Guo J, Currie G, Ceder A, Dong W, Pender B (2014) Bus bridging disruption in rail services with frustrated and impatient passengers. IEEE Trans Intell Transp Syst 15(5):2014–2023. 10.1109/TITS.2014.2307859

[CR315] Wang J, Yuan Z, Yin Y (2019) Optimization of bus bridging service under unexpected metro disruptions with dynamic passenger flows. J Adv Transp 2019:6965728. 10.1155/2019/6965728

[CR316] Weerawat W, Chumkad K (2018) A new operations approach for Bangkok Metro Green Line using short turn operation patterns. J Rail Transp Plan Manag 8(3):207–219. 10.1016/j.jrtpm.2018.06.001

[CR317] Wollnik M (1988) Ein Referenzmodell des Informationsmanagements. Inf Manag 3(3):34–43

[CR318] Wong YZ, Hensher DA, Mulley C (2020) Mobility as a service (MaaS): charting a future context. Transp Res Part A Policy Pract 131:5–19. 10.1016/j.tra.2019.09.030

[CR319] Wu J, Liu M, Sun H, Li T, Gao Z, Wang DZ (2015) Equity-based timetable synchronization optimization in urban subway network. Transp Res Part C Emerg Technol 51:1–18. 10.1016/j.trc.2014.11.001

[CR320] Wu W, Liu R, Jin W (2017) Modelling bus bunching and holding control with vehicle overtaking and distributed passenger boarding behaviour. Transp Res Part B Methodol 104:175–197. 10.1016/j.trb.2017.06.019

[CR321] Wu W, Liu R, Jin W (2018) Integrating bus holding control strategies and schedule recovery: simulation-based comparison and recommendation. J Adv Transp. 10.1155/2018/9407801 (Article ID 9407801)

[CR322] Wu W, Liu R, Jin W, Ma C (2019) Simulation-based robust optimization of limited-stop bus service with vehicle overtaking and dynamics: A response surface methodology. Transp Res Part E Logist Transp Rev 130:61–81. 10.1016/j.tre.2019.08.012

[CR323] Xie L, Naumann M, Suhl L (2012) A stochastic model for rota scheduling in public bus transport. In: Proceedings of the 2nd stochastic modeling techniques and data analysis international conference, Chania, Crete, pp 785–792. http://www.smtda.net/images/1_SMTDA2012_Proceedings_N-Z_557-802.pdf

[CR324] Xing Y, Lu J, Chen S, Dissanayake S (2017) Vulnerability analysis of urban rail transit based on complex network theory: a case study of Shanghai metro. Public Transp 9:501–525. 10.1007/s12469-017-0170-2

[CR326] Yamauchi T, Takamatsu M, Imahori S (2017) Optimizing train stopping patterns for congestion management. In: D’Angelo G, Dollevoet T (eds) 17th Workshop on algorithmic approaches for transportation modelling, optimization, and systems (ATMOS 2017). Open Access series in informatics (OASIcs), vol 59, pp 13:1–13:15. Schloss Dagstuhl–Leibniz-Zentrum für Informatik, Dagstuhl, Germany. 10.4230/OASIcs.ATMOS.2017.13

[CR325] Yamauchi T, Takamatsu M, Imahori S (2021) Optimizing train stopping patterns for congestion management. Public Transp. 10.1007/s12469-021-00286-w

[CR327] Yan Y, Meng Q, Wang S, Guo X (2012) Robust optimization model of schedule design for a fixed bus route. Transp Res Part C Emerg Technol 25:113–121. 10.1016/j.trc.2012.05.006

[CR328] Yan Y, Liu Z, Meng Q, Jiang Y (2013) Robust optimization model of bus transit network design with stochastic travel time. J Transp Eng 139(6):625–634. 10.1061/(ASCE)TE.1943-5436.0000536

[CR329] Yang J, Jin JG, Wu J, Jiang X (2017) Optimizing passenger flow control and bus-bridging service for commuting metro lines. Comput Aided Civ Infrastruct Eng 32(6):458–473. 10.1111/mice.12265

[CR330] Yao B, Hu P, Lu X, Gao J, Zhang M (2014) Transit network design based on travel time reliability. Transp Res Part C Emerg Technol 43:233–248. 10.1016/j.trc.2013.12.005

[CR331] Yap M (2014) Robust public transport from a passenger perspective: a study to evaluate and improve the robustness of multi-level public transport networks. Ph.D. thesis, Delft University of Technology, Delft

[CR332] Yap M, Cats O (2021) Public transport network resilience. In: Currie G (ed) Handbook of public transport research. Edward Elgar, Cheltenham, pp 226–251. 10.4337/9781788978668.00020

[CR333] Yap M, Nijenstein S, van Oort N (2017) Improving predictions of the impact of disturbances on public transport usage based on smart card data. In: Transportation Research Board 96th annual meeting. Washington DC

[CR334] Yap M, van Oort N, van Nes R (2018) Identification and quantification of link vulnerability in multi-level public transport networks: a passenger perspective. Transportation 45:1161–1180. 10.1007/s11116-018-9892-5

[CR335] Yin J, Tang T, Yang L, Gao Z, Ran B (2016) Energy-efficient metro train rescheduling with uncertain time-variant passenger demands: an approximate dynamic programming approach. Transp Res Part B Methodol 91:178–210. 10.1016/j.trb.2016.05.009

[CR336] Yin J, Tang T, Yang L, Xun J, Huang Y, Gao Z (2017) Research and development of automatic train operation for railway transportation systems: a survey. Transp Res Part C Emerg Technol 85:548–572. 10.1016/j.trc.2017.09.009

[CR337] Yin H, Wu J, Sun H, Qu Y, Yang X, Wang B (2018) Optimal bus-bridging service under a metro station disruption. J Adv Transp 2018:2758652. 10.1155/2018/2758652

[CR338] Yin H, Wu J, Liu Z, Yang X, Qu Y, Sun H (2019) Optimizing the release of passenger flow guidance information in urban rail transit network via agent-based simulation. Appl Math Model 72:337–355. 10.1016/j.apm.2019.02.003

[CR339] Yin H, Wu J, Sun H, Kang L, Liu R (2019) Optimizing last trains timetable in the urban rail network: social welfare and synchronization. Transportmatrica B Transp Dyn 7:473–497. 10.1080/21680566.2018.1440361

[CR340] Yu X, Li N (2020) How did Chinese government implement unconventional measures against COVID-19 pneumonia. Risk Manag Healthc Policy 13:491–499. 10.2147/RMHP.S251351 32581611 10.2147/RMHP.S251351 PMC7266822

[CR341] Yuan J, Gao Y, Li S, Liu P, Yang L (2022) Integrated optimization of train timetable, rolling stock assignment and short-turning strategy for a metro line. Eur J Oper Res 301:855–874 10.1016/j.ejor.2021.11.019

[CR342] Zeng AZ, Durach CF, Fang Y (2012) Collaboration decisions on disruption recovery service in urban public tram systems. Transp Res Part E Logist Transp Rev 48(3):578–590. 10.1016/j.tre.2011.11.005

[CR343] Zhan S, Kroon LG, Zhao J, Peng Q (2016) A rolling horizon approach to the high speed train rescheduling problem in case of a partial segment blockage. Transp Res Part E Logist Transp Rev 95:32–61. 10.1016/j.tre.2016.07.015

[CR344] Zhang S, Lo HK (2018) Metro disruption management: optimal initiation time of substitute bus services under uncertain system recovery time. Transp Res Part C Emerg Technol 97:409–427. 10.1016/j.trc.2018.11.001

[CR345] Zhang S, Lo HK (2020) Metro disruption management: contracting substitute bus service under uncertain system recovery time. Transp Res Part C Emerg Technol 110:98–122. 10.1016/j.trc.2019.11.010

[CR346] Zhang Y, Ng ST (2021) A hypothesis-driven framework for resilience analysis of public transport network under compound failure scenarios. Int J Crit Infrastruct Prot 35:100455. 10.1016/j.ijcip.2021.100455

[CR347] Zhang Y, Tang J (2018) A robust optimization approach for itinerary planning with deadline. Transp Res Part E Logist Transp Rev 113:56–74. 10.1016/j.tre.2018.01.016

[CR348] Zhang X, Li L, Le Vine S, Liu X (2019) An integrated pricing/planning strategy to optimize passenger rail service with uncertain demand. J Intell Fuzzy Syst 36:435–448. 10.3233/JIFS-181701

[CR349] Zhang S, Lo HK, Ng K, Chen G (2021) Metro system disruption management and substitute bus service: a systematic review and future directions. Transp Rev 41(2):230–251. 10.1080/01441647.2020.1834468

[CR350] Zhao S, Lu C, Liang S, Liu H (2016) A self-adjusting method to resist bus bunching based on boarding limits. Math Probl Eng. 10.1155/2016/8950209 (Article ID 8950209)

[CR351] Zieger S, Weik N, Nießen N (2018) The influence of buffer time distributions in delay propagation modelling of railway networks. J Rail Transp Plan Manag 8(3):220–232. 10.1016/j.jrtpm.2018.09.001

